# Integrated Taxonomy Discovers Four New Species of *Grypoctonus* Speiser, 1928 (Diptera: Asilidae) from China †

**DOI:** 10.3390/insects16070722

**Published:** 2025-07-15

**Authors:** Haoyue Zhou, Ding Yang, Xuankun Li

**Affiliations:** 1 State Key Laboratory of Agricultural and Forestry Biosecurity, College of Plant Protection, China Agricultural University, Beijing 100193, China; haoyue.zhou@cau.edu.cn (H.Z.); yangding@cau.edu.cn (D.Y.); 2 Department of Entomology, College of Plant Protection, China Agricultural University, Beijing 100193, China

**Keywords:** DNA barcoding, integrated taxonomy, new species, assassin fly

## Abstract

The genus *Grypoctonus* Speiser, 1928 (Diptera: Asilidae) is a fuzzy-looking assassin fly, and adults have only been observed in autumn and winter. Currently containing four described species, this genus is readily distinguished from other Chinese asilids by the presence of two *r-m* crossveins. Through integrative taxonomic analysis of over 200 specimens from multiple Chinese provinces, we combined morphological assessment with DNA barcoding and four species delimitation methods (ABGD, ASAP, mPTP, and GMYC). Four species are newly described: *G. aureus* **sp. nov.**, *G. sagittatus* **sp. nov.**, *G. solarius* **sp. nov.**, and *G. yongshani* **sp. nov.** (the latter described solely from morphological examination of historical specimens). Genetic analyses revealed distinct barcoding gaps, with an interspecific distance of 1.38–7.07% versus an intraspecific distance of no more than 0.92%. We revised the generic diagnosis, provided a distribution map, and a revised key to all known species of *Grypoctonus*.

## Simple Summary

*Grypoctonus* is a fuzzy-looking asilid genus currently containing four valid species distributed across the Palaearctic and high-elevation Oriental–Palaearctic transition zones. We freshly collected over 200 *Grypoctonus* specimens from nine sites across China between September 2023 and November 2024 and generated 164 COI barcodes using a MinION sequencing pipeline. We applied four species delimitation methods (ABGD, ASAP, mPTP, GMYC) alongside morphological evidence. Our results corroborated four species, which we describe here as new to science. Pairwise genetic distances of DNA barcode analyses revealed clear barcoding gaps. We revise the generic diagnosis, provide a global distribution map, include all known species, and offer an updated key.

## 1. Introduction

The genus *Grypoctonus* Speiser, 1928 [1] currently consists of four valid species: *G. aino* Speiser, 1928 [1], *G. lama* Speiser, 1928 [1], *G. hatakeyamae* (Matsumura, 1916 [2]), and *G. engeli* Hradský & Geller-Grimm, 1999 [3]. These species are primarily distributed across the Palaearctic region (China, Japan, Kazakhstan, Kyrgyzstan, Mongolia, Russia, and South Korea), with one record from the high-elevation Oriental–Palaearctic transition zone (Darjeeling, India; >2000 m elevation) [1,2,3,4,5]. *Grypoctonus* resembles *Cyrtopogon* Loew, 1847 [6] and *Pycnopogon* Loew, 1847 [6] but can be distinguished by the presence of two crossveins *r-m* (one crossvein in *Cyrtopogon* and *Pycnopogon*), cell *cua* open (closed in *Pycnopogon*), and a strongly protruded face (slightly protruded in *Pycnopogon*) [7]. Adults typically emerge from late August to early December, occupying a unique niche distinct from most other asilids.

Matsumura [2] described the first species of this genus (as *Pycnopogon hatakeyamae*). The genus *Grypoctonus* was established by Speiser [1] together with three newly described species, *G. aino*, *G. daimyo*, and *G. lama*. Engel [8], described the subspecies *G. daimyo chinensis* Engel, 1934, which was transferred to *Cyrtopogon* and treated as a species by Lehr [4]. In the illustrated handbook (*Iconographie Insectorum Japonicorum*) by Hisamatsu [9], *P. hatakeyamae* was placed in *Grypoctonus.* Lehr [10] considered *G. aino* as a junior synonym of *G. hatakeyamae*. The most recent revisionary work was conducted by Hradský & Geller-Grimm [3], which recovered *G. aino* as a validated species, synonymized *G. daimyo* into *G. hatakeyamae*, and described another new species, *G. engeli*, from India. Currently, three species of *Grypoctonus* have been recorded from China: *G. aino*, *G. hatakeyamae*, and *G. lama* [1,4,8,11,12].

The taxonomic status of *Grypoctonus* remains unresolved. It was placed in the subfamily Stenopogoninae by Hull [7] and Geller-Grimm [13] but was unplaced in the classification system by Dikow [14]. Species of *Grypoctonus* were not sampled in recent phylogenetic studies [14,15,16]. *Grypoctonus* used to be treated as a junior synonym of *Cyrtopogon* [4,8,11,17,18,19] but was not followed by subsequent studies.

In this study, we describe four new species of *Grypoctonus* from China, integrating morphological characters and molecular data with different species delimitation methods. The generic diagnosis is revised, and a distribution map and a revised key to the world species of *Grypoctonus* are provided.

## 2. Materials and Methods

### 2.1. Fieldwork

Two hundred forty-three specimens of *Grypoctonus* and one *Cyrtopogon centralis* Loew, 1871 [20] were freshly collected from five sites in Beijing, two sites each in Yunnan and Hebei Provinces, and one site each in Inner Mongolia Autonomous Region and Henan Province between September 2023 and November 2024. Geographical coordinates were obtained using the cell phone app 2bulu (https://www.2bulu.com/, accessed on 6 July 2025), and coordinates of new collection sites are listed in Supplementary File S1. All specimens were collected alive by a hand net. Specimens were killed in a −20 °C freezer or ethyl acetate killing jar, and the right hind legs were immediately removed and preserved in 95% ethanol. These tissue samples were maintained at −20 °C in the laboratory until DNA extraction. The remaining specimens were mostly pinned or preserved in 95% ethanol and maintained at −20 °C.

### 2.2. Morphological Study

Photos of specimens were taken using a Canon 5D mark III digital camera (Canon Co., Ltd., Beijing, China) with an RF 24–105 mm F4 L IS USM lens. Genitalia were prepared by heating the whole abdomen in 10% NaOH solution at 56 °C for 3.5 h using the JOANLAB DB100-2P metal bath (Joan Lab Equipment Co., Ltd., Zhejiang, China) to clear needless fats and tissues, and they were kept in the same liquid at room temperature with their status checked regularly. After rinsing the NaOH with ultrapure water, the genitalia were transferred to glycerine for examination and to KY lubricant for photography. Female genitalia were dyed with 1% chlorazol black solution for 30 s to 1 min, placed in eosin Y solution, microwaved for 4 s, and let stand for 15 min after rinsing the chlorazol black with water. The genitalia were photographed by Leica DM2000 (Leica Microsystems, Wetzlar, Germany) fitted with a Nikon D850 digital camera (Nikon (China) Corporation, Shanghai, China), and the residual NaOH was neutralized with 30% CH_3_COOH solution. Genitalia were preserved in a 0.2 mL tube containing a 70% glycerine and 30% ethanol mixture with Bemis Parafilm at room temperature. All photos were superimposed by Helicon Focus v.7.6.3 with default Method C settings (pyramid), and the genitalia photos were processed with Topaz Mask AI v.1.3.9 to keep the surroundings clean. Morphological terminology mainly follows Londt and Dikow [21], Cumming and Wood [22], and Dikow [14].

### 2.3. DNA Extraction, PCR, and Sequencing

Our molecular work mainly followed the MinION barcoding pipeline by Srivathsan and Meier [23] with some modifications. In short, DNA was extracted using the HotSHOT (Truett et al. [24]) method with 10 μL (for smaller species) or 25 μL (for larger species) alkaline lysis buffer and neutralization buffer per leg in 96-well plates. We modified the universal primers to better match Diptera, the F-primer (5′-TAAACTTCTGGATGTCCAAAAAATCA-3′), and the R-primer (5′-TTTCAACAAATCATAAAGATATTGG-3′), with 13 bp dual-indexed tags added on the end of each primer. Each PCR reaction consisted of 10 μL 2× Es Taq MasterMix (CWBio), 5 μL ddH_2_O, 3 μL DNA, and 1 μL each of primer (10 μM). PCR amplification conditions used a 5 min initial denaturation at 95 °C, followed by 35 cycles of a 1 min denaturation at 94 °C, 2 min annealing at 45 °C, and 1 min extension at 72 °C, followed by 5 min final extension at 72 °C. A subset of 16 products per plate was run in 1% agarose gels to assess PCR success. A total of 6 μL of PCR products per well were pooled and purified using SPRIselect beads (Beckman Coulter, Inc., Brea, CA, USA). DNA concentrations were measured using an ALLSHENG Fluo-200 fluorometer (Hangzhou Allsheng Instruments Co., Ltd., Hangzhou, China) with the dsDNA HS Assay Kit (Qubit) (Thermo Fisher Scientific Inc., Shanghai, China). Library preparation was performed using the SQK-LSK114 Ligation Sequencing Kit (Oxford Nanopore Technologies, Oxford, England), NEB#E7546S Ultra II End Repair Ligation Module (New England Biolabs Ltd., Beijing, China), and NEB#E6056S Quick Ligation Module (New England Biolabs). The final library was sequenced using a MinION Mk1B device with the MinION R10.4.1 flow cell and MinKNOW v.24.06.16. DNA sequences were demultiplexed using ONTbarcoder (Srivathsan et al. [25]).

### 2.4. Molecular Species Delimitation

*Cyrtopogon centralis* Loew was used as an outgroup. Four molecular species delimitation methods were used. We performed ABGD analysis (Puillandre et al. [26]) using the iTaxotools bioinformatics platform (Vences et al. [27]) with Pmin = 0.001, Pmax = 0.1, steps = 20, X = 1.5, and K2P distance. We performed ASAP analysis with K2P distance and default parameters; the smallest ASAP score is the best partition, which we considered as the result (Puillandre et al. [28]). We generated a maximum likelihood (ML) tree using IQ-TREE v.2.4.0 (Minh et al. [29]) under the GTR model (Tavaré [30]). We performed mPTP (Kapli et al. [31]) analysis on the web server (https://mptp.h-its.org/ accessed on 10 March 2025) without the crop outgroup and used the ML tree as the input file. We generated the ultrametric tree using BEAST v.1.10.4 (Suchard et al. [32]) on the high-performance computing clusters at the China Agricultural University. The data matrix was imported into BEAUti (Drummond et al. [33]). An uncorrelated relaxed clock with a lognormal distribution was applied, with the HKY substitution model and estimated base frequencies, and the site heterogeneity was modeled with gamma + invariant sites. A birth–death process was used as the tree prior, with the operator mix set to the classic operator mix. The Markov chain was run for 200,000,000 generations, sampling every 5000 generations. TRACER v.1.7.2 (Rambaut et al. [34]) was used for checking whether all effective sample size (ESS) values exceeded 200 for assessing the convergence of runs. We generated a consensus tree after a burn-in of 25% using TreeAnnotator v.1.10.4 (BEAST package). We saved the ultrametric trees in Newick format and utilized ape (Paradis et al. [35]) and splits (Joseph & Vakayil [36]) packages to run GMYC (Pons et al. [37]) analysis. Based on the species hypotheses provided by morphology and different molecular species delimitation methods, we calculated the inter- and intraspecific distances. Pairwise genetic distances of DNA barcodes were calculated in MEGA v.11.0 (Tamura et al. [38]) based on the Kimura 2-parameter (K2P) model (Kimura [39]) with transitions + transversions substitutions, uniform rates, pairwise deletion, and selecting all codon positions.

### 2.5. Distribution Map

The distribution map was generated with QGIS Desktop v. 3.40.4 (QGIS Development Team [40]). Topographic shading and Application Programming Interface URL were cited in the Chinese National Platform for Common GeoSpatial Information Services (www.tianditu.gov.cn accessed on 15 Mar 2025). The SRTMDEM 90M resolution raw elevation data was downloaded in the Geospatial Data Cloud (www.gscloud.cn accessed on 15 Mar 2025).

### 2.6. Abbreviations

#### 2.6.1. Collections

CAU = Entomological Museum, China Agricultural University, Beijing, China.
EIHU = Entomological Institute, Hokkaido University, Sapporo, Japan.
MWNH = Museum Wiesbaden, Wiesbaden, Germany.
NACRC, IOZ = National Animal Collection Resource Center, Institute of Zoology, Chinese Academy of Sciences, Beijing, China.
ZSM = Zoologische Staatssammlung München (Bavarian State Collection of Zoology), München, Germany.

#### 2.6.2. Localities

Ngawa = Ngawa Tibetan and Qiang Autonomous Prefecture.
Weichang = Weichang Manchu and Mongol Autonomous County.
Diqing = Diqing Tibetan Autonomous Prefecture.
Garzê = Garzê Tibetan Autonomous Prefecture.

## 3. Results

### 3.1. DNA Barcode-Based Species Delimitation

**Figure 1:**
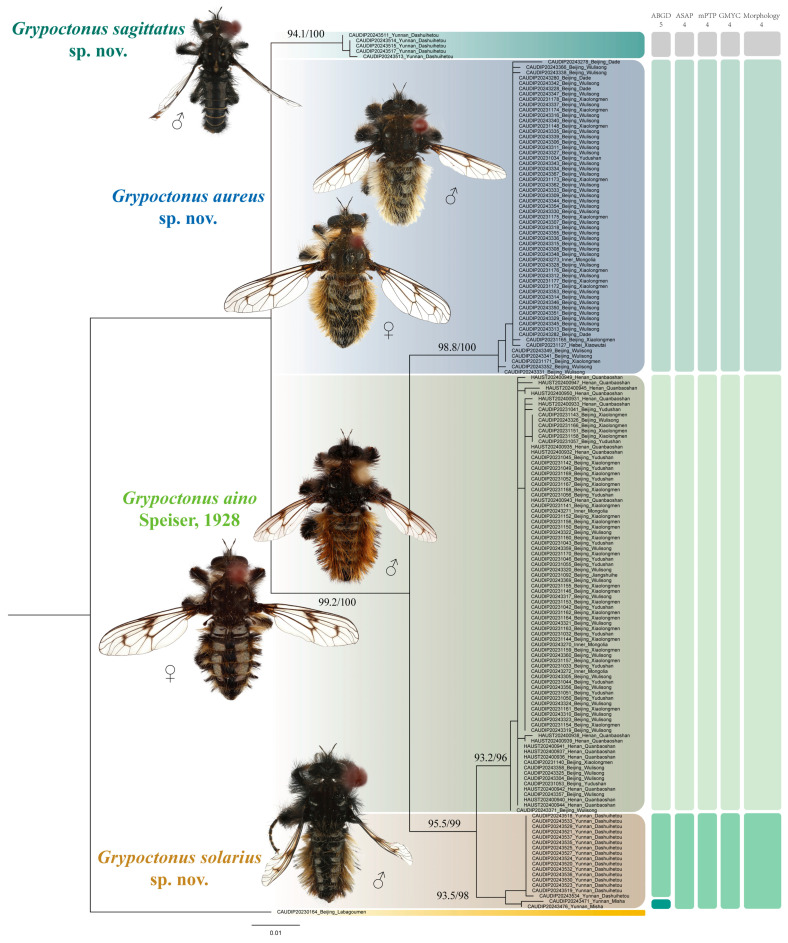
Integrative species delimitation of *Grypoctonus* combining molecular and morphological evidence. COI phylogeny was generated by the maximum likelihood method, branch lengths were scaled to the substitution rates, and branch supports (SH-aLRT/UFBoot values) were only for species-level and higher clades. The results from four delimitation methods (ABGD, ASAP, mPTP, and GMYC) are shown alongside morphological assessments.

**Figure 2:**
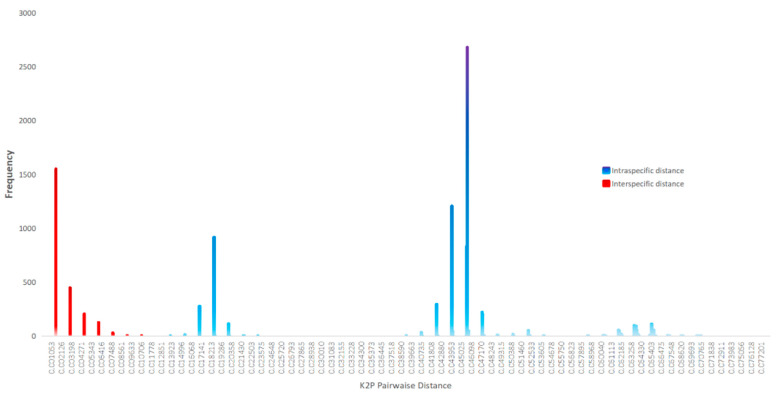
Pairwise distance of four *Grypoctonus* species under the K2P model, including *G. aino*, *G. solarius* **sp. nov.**, *G. aureus* **sp. nov.**, and *G. sagittatus* **sp. nov.**

We yielded 164 COI sequences of *Grypoctonus* and one COI sequence of *Cyrtopogon centralis* Loew as an outgroup; all sequences are deposited on the BOLD system (Supplementary File S2). All sequences comprised the full 658 bp barcode region. The results of ASAP, mPTP, and GMYC were consistent with the morphological result that supported four species, while ABGD analysis suggested five species by separating the Misha population and Dashuihetou population of the *G. solarius* **sp. nov.** (Figure 1). Based on a comprehensive morphological assessment and majority support from other species delimitation methods, we maintain these populations as conspecific. Genetic distance analyses revealed clear barcoding gaps between intra- and interspecific variation (Figure 2). Interspecific distances ranged from 1.38% to 7.07%, with the lowest observed between *G. aino* and *G. solarius* **sp. nov.** (1.38–2.32%). Intraspecific distances were substantially lower (no more than 0.92%), showing no overlap with interspecific distance (Figure 2; Supplementary File S3). For the four species with COI sequences, the number of COI sequences/number of species was as follows: *G. aino* (*n* = 82/104), *G. aureus* (*n* = 59/74), *G. solarius* (*n* = 18/53), and *G. sagittatus* (*n* = 5/12).

### 3.2. Taxonomy

Family **Asilidae** Latreille, 1802.

**Genus *Grypoctonus* Speiser, 1928.**

*Grypoctonus* Speiser, 1928: 155 [1]. Type species: *Grypoctonus aino* Speiser 1928: 156, by original designation.

Chinese common name: 辅脉食虫虻属 (following Hua, 1990 [41]).

**Notes.** Two Chinese common names exist for this genus. “辅脉食虫虻属” is derived from the second crossvein *r-m* and was named by Hua [41], and it was followed by Yu & Wang [42]. “驼食虫虻属” comes from a literal translation of “Grypo–” and was proposed by Zhang & Yang in Yang et al. [12]. We decided to use the first Chinese name as it has a longer history and is more widely accepted.

**Diagnosis.** Body covered in long hairs. Face entirely protruding and covered with long hairs. Stylus two-segmented, with apical seta-like sensory article. Wing with two crossveins *r-m*. Male epandrium largely separated. Hypandrium with mammillary processes. Inner and outer apex of gonocoxite fingerlike and tapered. Female acanthophorite spines present. Sperm pump transparent, slender, and fusiform. Spermatheca coiled entirely, spiral-shaped.

#### 3.2.1. Generic Redescription

**Original description by Speiser (1928):** “*Nächste Verwandtschaft mit* Cyrtopogon *Lw., unbedingt abweichend durch eine ungewöhnliche Querader über dem Ende der Discoidalzelle zwischen den Längsadern r und m, so daß die Erste Hinterrandzelle in zweie geteilt ist. r*_4+5_ *von der Kleinen Querader bis zur Gabelung mindestens doppelt so lang wie die Gabel selber (bei* Cyrtopogon *nur ebenso lang wie die Gabel). Die Analis verläuft nicht gerade, sondern ist an ihrem Ende mit einem plötzlichen Bogen randabwärts gebogen, so daß die auch hier offene Analzelle an ihrem Ende etwa schnabelartig gestaltet ist. Behaarung erheblich stärker als bei* Cyrtopogon*, ausgesprochen zottig, auch auf den Beinen, und besonders den Vorder- und Mittelbeinen. Typus generis:* G. aino *nov. spec*.”

**English Translation:** Closesly related to *Cyrtopogon* Loew, but distinctly differing by an unusual crossvein beyond the end of the discal cell between the longitudinal veins *r* and *m*, thereby dividing the first posterior cell into two parts. *r*_4+5_ from the small crossvein to the fork is at least twice as long as the fork itself (in *Cyrtopogon*, it is only as long as the fork). The anal vein does not run straight but curves abruptly downward toward the margin at its end, giving the open anal cell *a* somewhat beak-like shape at its tip. The pubescence is significantly denser than in *Cyrtopogon*, distinctly shaggy, including on the legs—especially the forelegs and midlegs. Type species of the genus: *G. aino* **sp. nov.**

**Redescription.** Middle- to small-sized Asilidae, body length 8–15 mm, wing length 9–20 mm, body covered in long hairs.

**Head.** Face entirely protruding and covered with long hairs. Ocellar tubercle with long hairs. Stylus two-segmented, apical segment longer than basal segment, with apical seta-like sensory article. Palpus clavate, two-segmented.

**Thorax.** Antepronotum, postpronotum, proepimeron, postalar callus, anepisternum, katepisternum, metanepisternum, katatergite, scutum, and scutellum with hairs. Anepimeron, katepimeron, metakatepisternum, and meron bare. Scutum with middle strip.

**Legs.** Ventral face of fore tibia with dense short hairs; posterior face on hind tibia with dense long hairs.

**Wings.** Wing membrane hyaline with infuscated in wing base and middle area (usually concentrate on crossvein *r-m*_1_, *r-m*_2_, base of cell *m*_1_, posterior of cell *bm,* and apex of cell *br*). Two crossveins *r-m* present; cell *r*_1_ open, vein *R*_4_ curved forward and reach to wing margin before the apex of wing, cells *m*_3_, and *cua* open; and vein *C* circumambient, base with a row of dense, short, and robust hairs. Alula vestigial.

**Abdomen.** Dark metallic with long hairs. **Genitalia. Male.** Epandrium relatively short, trapezoid, and largely separated, only connected at the anterior margin; anterior margin strongly concaved. Hypandrium large, trapezoid. and fused with gonocoxite; anterior margin convex, with one pair of mammillary processes extending from the posterior margin. Gonocoxite widely separated, each side subtriangular from ventral view; ejaculatory apodeme narrow, not extending anterior margin of hypandrium; lateral ejaculatory process present, triangles. Gonocoxal apodeme present, not extending anterior margin of hypandrium. Inner apex of gonocoxite subtrianglar, fingerlike with hairs apically from ventral view. Outer apex of gonocoxite fingerlike, ventrally pointed, and apex tapered. Gonocoxite with large ventral extension on posterior half. Gonostylus fingerlike, posterior pointed, and apex slightly curved dorsally. Dorsal aedeagal sheath trifurcated or blunt, ventral aedeagal sheath bifid or four-branched. **Female.** Tergite 9 + 10 divided in the middle, acanthophorite spines on each side located on two plates, one above cerci, another on ventrolateral of cerci (these two plates might be secondary divisions of T9 + 10 rather than T9 and T10 but need to be compared with closely related genera). Genital fork connected anteriorly, U-shaped. Sperm pump present, transparent, relatively slender and fusiform, collars absent. Distal spermathecal duct transparent and slender, around 4× longer than sperm pump. Spermatheca large, coiled entirely, spiral-shaped.

#### 3.2.2. Species Description

***Grypoctonus aino* Speiser, 1928.**

**Figure 3:**
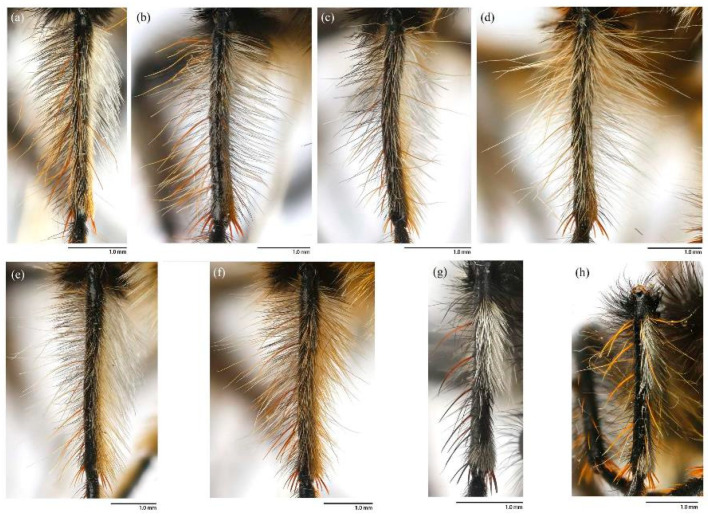
Posterior face of hind tibia of *Grypoctonus* species. (**a**) *G. aino* Speiser, 1928, male; (**b**) *G. aino* Speiser, 1928, female; (**c**) *G. solarius* **sp. nov.**, male; (**d**) *G. solarius* **sp. nov.**, female; (**e**) *G. aureus* **sp. nov.**, male; (**f**) *G. aureus* **sp. nov.**, female; (**g**) *G. sagittatus* **sp. nov.**, male; (**h**) *G. Yongshani* **sp. nov.**, male.

**Figure 4:**
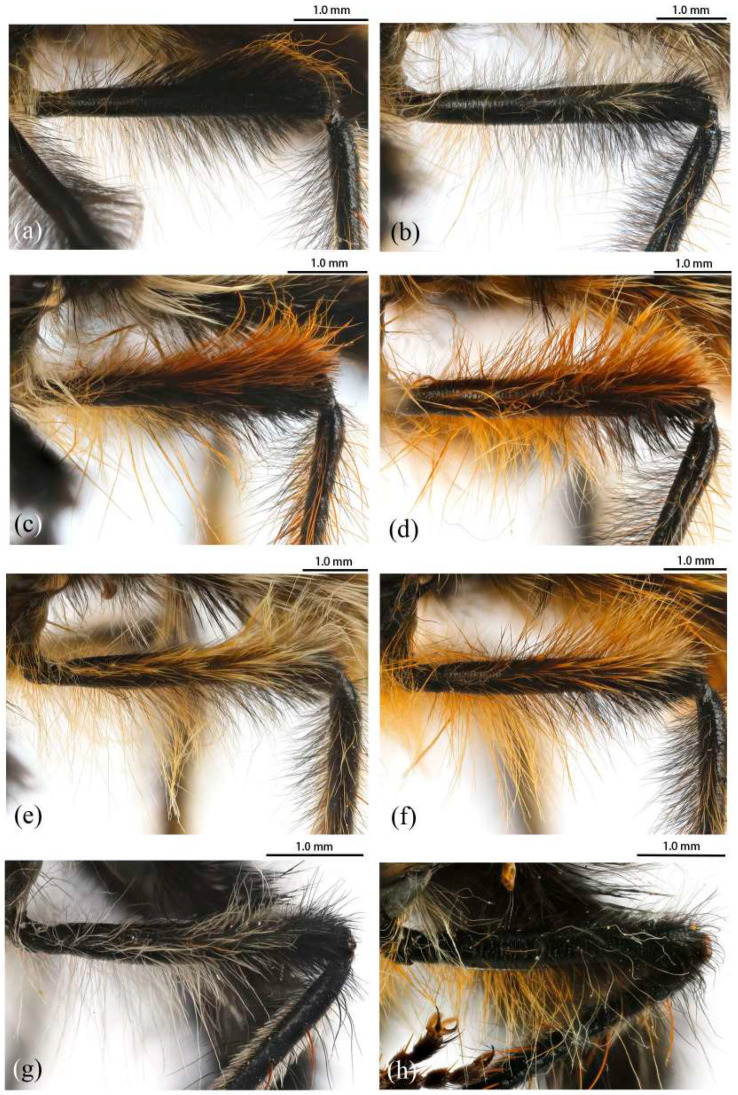
Anterior face of hind femora of Grypoctonus species. (**a**) *G. aino* Speiser, 1928, male; (**b**) *G. aino* Speiser, 1928, female; (**c**) *G. solarius* **sp. nov.**, male; (**d**) *G. solarius* **sp. nov.**, female; (**e**) *G. aureus* **sp. nov.**, male; (**f**) *G. aureus* **sp. nov.**, female; (**g**) *G. sagittatus* **sp. nov.**, male; (**h**) *G. yongshani* **sp. nov.**, male.

**Figure 5:**
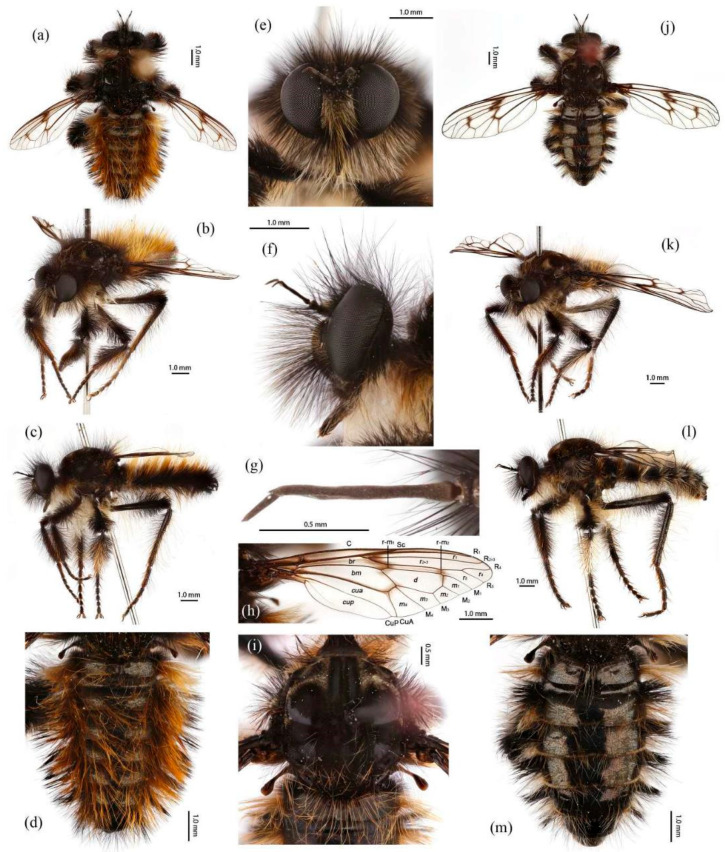
*Grypoctonus aino* Speiser, 1928, adult. (**a**–**i**) Male; (**j**–**m**) female. (**a**) Body, dorsal; (**b**) body, profile; (**c**) body, lateral; (**d**) abdomen, dorsal; (**e**) head, frontal; (**f**) head, lateral; (**g**) antenna, lateral; (**h**) wing; (**i**) thorax, dorsal; (**j**) body, dorsal; (**k**) body, profile; (**l**) body, lateral; (**m**) abdomen, dorsal.

**Figure 6:**
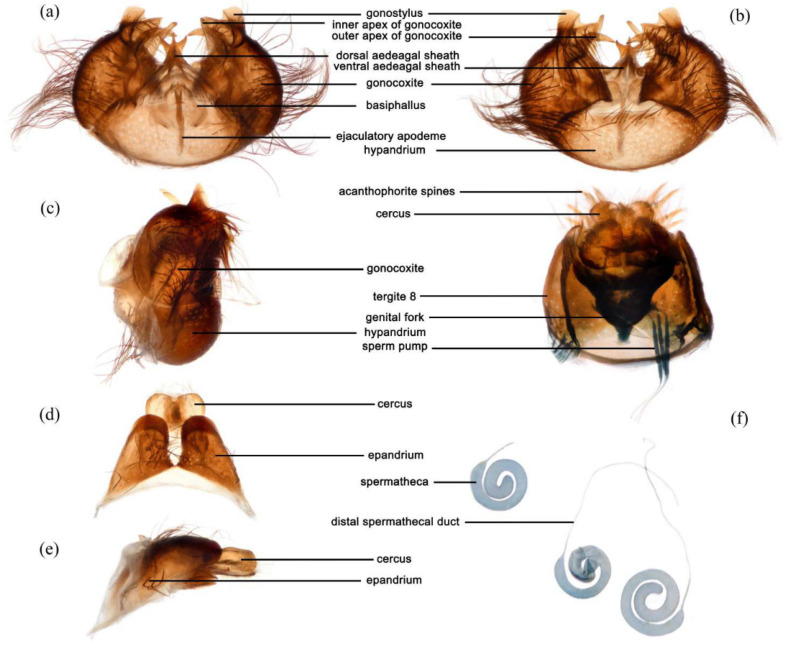
*Grypoctonus aino* Speiser, 1928, genitalia. (**a**–**c**) Male gonocoxite and hypandrium; (**d**–**e**) cercus and epandrium. (**a**) Dorsal; (**b**) ventral; (**c**) lateral; (**d**) dorsal; (**e**) lateral; (**f**) female genitalia ventral.

**Figure 17:**
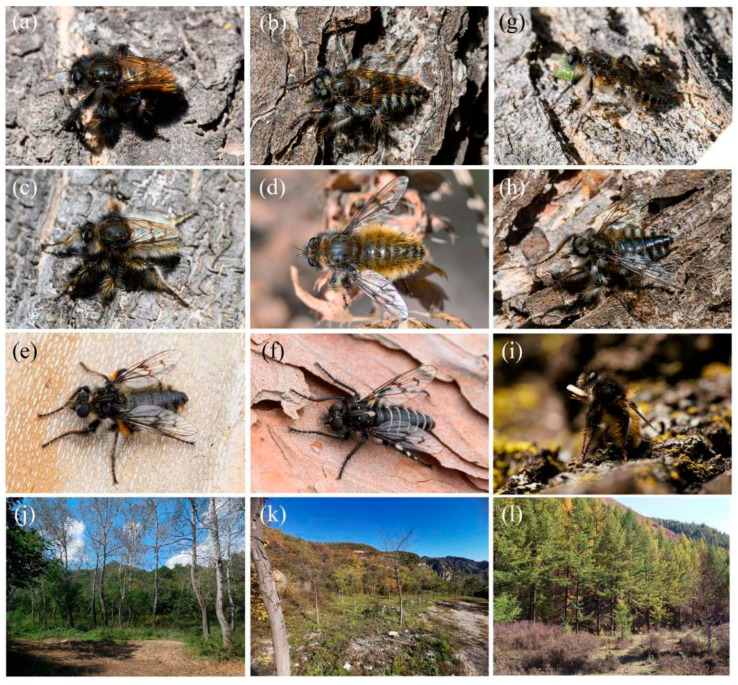
Living photos, habitats, and biology of the genus *Grypoctonus*. (**a**) *G. aino* Speiser, 1928, male (photoed by Tianyu Zheng); (**b**) *G. aino* Speiser, 1928, female (photographed by Tianyu Zheng); (**c**) *G. aureus* **sp. nov.**, male (photographed by Tianyu Zheng); (**d**) *G. aureus* **sp. nov.**, female (photographed by Tianyu Zheng); (**e**) *G. solarius* **sp. nov.**, female (photographed by Tianyu Zheng); (**f**) *G. sagittatus* **sp. nov.**, female, (photographed by Tianyu Zheng); (**g**) *G. aino*, female, predation [Miridae] (photographed by Tianyu Zheng); (**h**) *G. aino,* female, predation [Aphidoidea] (photographed by Tianyu Zheng); (**i**) *G. aino*, male, predation [Cicadellidae] (photographed by Yuezheng Tu); (**j**) habitats in Yudu Mt., Beijing, September; (**k**) habitats in Wulisong, Beijing, October; (**l**) habitats in Sumu Mt., Inner Mongolia, October (photographed by Zhanquan Ling).

Chinese common name: 爱辅脉食虫虻 (following Yu & Wang [42]).

(Figure 3a,b, Figure 4a,b, Figure 5, Figure 6, and Figure 17a,b,g–i).

*Grypoctonus aino* Speiser, 1928: 156. Type locality: Japan, Nagasaki; holotype in ZSM, 4 ♂.

Engel, 1930: 324 (key; description; wing) [43]; Engel, 1934: 13 (specimen information; description; *Cyrtopogon*) [8]; Bromley, 1945: 93 (cat.; *Cyrtopogon*) [18]; Aoki, 1950: 1600 (drawing; description; distribution; *Cyrtopogon*) [17]; Hull, 1962: 184 (list) [7]; Hisamatsu, 1965: 202 (description; distribution) [9]; Hradský & Geller-Grimm, 1999: 100 (**stat. rev.**; key; specimen information; genitalia; description) [3]; Harusawa, 2002: 13 (distribution; biology) [44]; Harusawa, 2004 (distribution; biology) [45]; Young, 2005: 102 (key; description; male gentalia; distribution) [46]; Zhang & Yang in Yang et al., 2018: 121 (cat.; distribution) [12]; Tagawa, 2020 (photo) [47]; Yu & Wang, 2023: 54 (photo; description; distribution) [42]; Bock & Mengual, 2023: 80 (cat.; photo) [48].

**Materials examined. CHINA: BEIJING:** 12 ♂♂ 7 ♀♀, Yanqing [延庆], Yudushan Scenic Area [玉渡山风景区], 40.55° N, 115.88° E, 887 m, 21.IX.2023, Xuankun Li, Yuezheng Tu, Haoyue Zhou (CAU) (CAUDIP20231032–CAUDIP20231033, CAUDIP20231041–CAUDIP20231057); 3 ♂♂ 27 ♀♀, Mentougou [门头沟], Xiaolongmen National Forest Park [小龙门国家森林公园], 39.96° N, 115.43° E, 1150 m, 24.X.2023, Xuankun Li, Yuezheng Tu, Haoyue Zhou, Yan Lai (CAU) (CAUDIP20231139–CAUDIP20231147, CAUDIP20231149–CAUDIP20231164, CAUDIP20231166–CAUDIP20231170); ♂, Mentougou [门头沟], Qingshui [清水镇], Jiangshuihe [江水河村], 40.04° N, 115.50° E, 1550 m, 4.X.2023, Yuezheng Tu (CAU) (CAUDIP20231092); 13 ♂♂ 10 ♀♀, Changping [昌平], Wulisong [五里松], 40.19° N, 115.88° E, 905 m, 11.X.2024, Haoyue Zhou, Zhanquan Ling, Hongna Guo, Dong Guo, Tianyu Zheng (CAU) (CAUDIP20243302, CAUDIP20243304–CAUDIP20243305, CAUDIP20243310, CAUDIP20243317, CAUDIP20243319–CAUDIP20243326, CAUDIP20243356–CAUDIP20243361, CAUDIP20243368–CAUDIP20243371); ♂, Fangshan [房山], Baihua Mt. [百花山], 1300 m, 21.IX.1984, Long Yang (NACRC, IOZ). **HEBEI:** 2 ♂♂ 4 ♀♀, Chengde [承德], Weichang [围场县], Saihanba National Forest Park [塞罕坝国家森林公园], 42.39° N, 117.30° E, 1599 m, 15.X.2024, Wei Xu, Tao Li (CAU). **HENAN:** 10 ♂♂ 12 ♀♀, Luoyang [洛阳], Luoning [洛宁县], Xiayu [下峪镇], Quanbao Mt. [全宝山], 34.12° N, 111.41° E, 1225 m, 6.XI.2024, Hongna Guo, Hao Liu, Xiaohan Ye, Bei Zhou, Yunzhu Huo (CAU) (HAUST202400930–HAUST202400951). **INNER MONGOLIA:** 1 ♂ 3 ♀♀, Ulanqab [乌兰察布], Xinghe [兴和县], Sumu Mt. [苏木山], 40.34° N, 113.46° E, 1830 m, 4.X.2024, Tianyu Zheng, Zhanquan Ling (CAU) (CAUDIP20243269–CAUDIP20243272, CAUDIP20243274–CAUDIP20243275). **SICHUAN:** 1 ♂ 1 ♀, Ngawa [阿坝], Jiuzhaigou [九寨沟], 2300 m, 6.IX.1983, Xuezhong Zhang (NACRC, IOZ).

**Diagnosis. Male.** Anterior and dorsal face of hind femora mostly with black hairs, but dorsal face admixed with several dark orange hairs. Anepisternum densely covered in long black hairs, hairs denser posteriorly and dorsally; Katatergite densely covered in long black hairs, admixed with long golden yellow hairs. Abdominal tergites with denser long black hairs posterolaterally and golden yellow hairs dorsally. Dorsal aedeagal sheath trifurcated, branches long, with the same length. **Female.** Anepisternum densely covered in long black hairs admixed with long pale hairs posteriorly. Scutum with sparse black hairs. Katatergite densely covered in long dark yellow hairs, admixed with black hairs; Tergites almost cover with black hairs and white hairs laterally.

**Description. Male.** Body length 9–12 mm, wing length 14–18 mm.

**Head.** Head about 1.5× wider than high (frontal view) (Figure 5e) and 1.4× higher than long (lateral view, including face) (Figure 5f), mostly black with light yellow pruinescence and covered in dense long black hairs with admixed long, white to light yellow hairs. Frons trapezoid with light yellow pruinescence, 1.1× length of ocellar tubercle, 2.9× as wide as ocellar tubercle, with black hairs (hairs relatively short and sparse compared to hairs on other parts of the head). Ocellar tubercle slightly raised, black with sparse light yellow pruinescence, with long black hairs. Face with thick light yellow pruinescence and admixed dense long black and yellow hairs (yellow hairs denser on dorsal half), except dorsolateral area with narrow, bare and shinning strip (Figure 5e,f). Mystax not distinguishable from facial hairs. Gena narrow, with sparse light yellow pruinescence. Clypeus with thick light yellow pruinescence and otherwise bare. Occiput with thick light yellow pruinescence and dense long black hairs, ventral half with admixed dense long white to light yellow hairs, hairs denser in ventral half. Antenna black with sparse light yellow pruinescence, uniform from base to apex, scape and pedicel with dense long black hairs, flagellum bare (Figure 5g). Scape 1.5× as long as wide, and 1.2× as long as pedicel; pedicel 1.2× as long as wide; flagellum 10.5× as long as wide, 2.0× as long as scape + pedicel, 3.6× as long as scape; stylus 0.3× as long as flagellum, two-segmented, with apical seta-like sensory article; apical segment 4.0× as long as basal segment. Palpus clavate, black with fine brownish to blackish hairs, two-segmented. Proboscis black, only slightly compressed laterally, basal 2/3 with sparse pruinescence and dense long light yellow hairs ventrally, apical 1/3 shinning, with short yellow hairs.

**Thorax.** Integumental color of scutum mostly brownish black with light yellow pruinescence, scutum with brown middle strip reaching posterior 4/5 of the scutum, anterolateral of transverse suture and inner-anterior of postalar callus with small, bare, and shinning spots (Figure 5i). Antepronotum with admixed long light yellow and black hairs; postpronotum with long black hairs laterally; proepimeron densely covered in long white to pale yellow hairs. Lateral postpronotal lobe with shiny and dirty orange spot. Scutum covered with dense brown pruinescence and sparse long black hairs. Scutellum black with sparse brown pruinescence, covered with dense long golden yellow hairs admixed few black hairs, hairs in anterior margin strongly proclinate. Postalar callus with dense long black hairs. Pleura black with thick brown pruinescence; anepisternum densely covered in long black hairs, hairs denser posteriorly and dorsally; katepisternum with sparse black and light yellow hairs on dorsal margin; anepimeron, katepimeron, and meron bare. Metanepisternum covered in brown and pale yellow tomentums in middle area, posteroventral with sparse black hairs; metakatepisternum bare; katatergite densely covered in long black hairs, admixed with long golden yellow hairs; anatergite covered with dense light yellow pruinescence on dorsal margin.

**Legs.** Legs black, coxae covered in dense light yellow and white hairs, femora mostly covered in dense long black hairs, ventral face admixed with dense long light yellow hairs. Dorsal half of mid and hind femora admixed with a few dark yellow hairs; ventral face of hind femora admixed with light yellow and white hairs (Figure 4a). Fore tibiae mostly covered in dense long black hairs and strong long dark orange bristles, ventral face of fore tibia with dense short dark orange hairs on apical 4/5; dorsal face of mid tibia with black hairs, admixed with long yellow hairs and short white hairs; hind tibia posterior face with dense long white hairs on basal 4/5 and ventral face with dense short golden yellow hairs, admixed with long dark orange hairs (Figure 3a). Ventral and a part of posterior face of tarsi with dense short dark orange or golden yellow hairs; tarsi with short, robust black hairs and with dark orangish bristles. Fore tibia 2.8× longer than fore basitarsus, mid tibia 3.1× longer than mid basitarsus, hind tibia 3.0× longer than hind basitarsus.

**Wings.** Wing membrane hyaline with infuscated wing base, anterior margin, apex and middle of cell *br*, apex and posterior of cell *bm*, and areas around crossveins *r-m*_1_, *r-m*_2_, and base of cell *m*_1_. Haltere stem and knob black (Figure 5h).

**Abdomen.** Integumental color of tergites black mostly with dark blue metallic and sparse light yellow pruinescence (Figure 5d). Tergite 1 with dense long dark orange and golden yellow hairs, laterally admixed with more white hairs, and anterolaterally covered in dense short black hairs; tergites 2–6 with dense long dark orange and golden yellow hairs, laterally with dense long black hairs; tergites 7–8 with long black hairs, tergite 7 admixed with few dark yellow hairs. Sternites black with thick light yellow pruinescence, covered in long golden hairs admixed with some short black hairs; sternite 1 with yellow hairs, sternite 2 with black hairs, other sternites admixed with black and yellow hairs. Genitalia (Figure 6a–e). Posterior margin of epandrium 1.2× longer than full length, the length between middle point of posterior and anterior margins 0.6× longer than full length. Basiphallus enlarged. Outer apex of gonocoxite long. Gonocoxite with large ventral extension on posterior half, bifid, blunt posteriorly, tapered innerly. Dorsal aedeagal sheath trifurcated, branches long, with the same length.

**Female.** Body length 8–14 mm, wing length 14–21 mm. Distinctly different from male. Overall hairs sparser and with more paler hairs than black hairs. **Head.** About 1.6× wider than high (frontal view) and 1.3× higher than long (lateral view, including face). Frons 1.1× higher than ocellar tubercle, 2.5× as wide as ocellar tubercle. Ocellar tubercle with long light yellow to white hairs. Face with thick light yellow pruinescence. Scape 1.7× as long as wide, and 1.6× as long as pedicel; pedicel 1.0× as long as wide; flagellum 11.7× as long as wide, 2.2× as long as scape + pedicel, 3.6× as long as scape; stylus 0.2× as long as flagellum; apical segment 1.6× as long as basal segment.

**Thorax.** Postpronotum with long black and pale hairs laterally; anepisternum densely covered in long black hairs dorsally and admixed long pale hairs posteriorly; postalar callus admixed with black and yellow hairs; katatergite densely covered in long dark yellow hairs, admixed with black hairs.

**Legs.** Femora mostly covered in dense long black hairs, admixed with light yellow and white hairs; posterior face of fore femora admixed with long black and white hairs, ventral face of fore femora with denser long light yellow hairs; anterior face and basal 4/5 of ventral face of mid and hind femora admixed with light yellow to white hairs (Figure 4b). Dorsal face of mid and hind tibia with dense long white hairs admixed with dark orange hairs (Figure 3b). Fore tibia 2.6× longer than fore basitarsus, mid tibia 3.5× longer than mid basitarsus, hind tibia 2.9× longer than hind basitarsus.

**Wings.** Anterior half of wing membrane less infuscated, especially in cells *c* and *sc* (Figure 5j).

**Abdomen.** Tergites covered in sparse golden yellow hairs, admixed with short black and white hairs, laterally with dense, long black and white hairs. Tergite 1 with dense long yellow hairs and anterolaterally covered in dense short black hairs; tergites 2–6 laterally with dense long black and white hairs; tergites 7–8 with long yellow hairs, tergite 7 admixed with few black hairs (Figure 5m). Genitalia. Acanthophorite spines located on plate above cerci with six spines on each side, on plate ventrolateral of cerci with four larger spines and several smaller spines (Figure 6f).

**Remarks.** In some examined specimens, the apex of *R*_4+5_ was infuscated lightly; the female abdomen had denser yellow hairs.

**Distribution. China:** Beijing (Yu & Wang, 2023 [42]; this paper); Gansu (Engel, 1934 [8]); Hebei (newly recorded); Henan (newly recorded); Inner Mongolia (newly recorded); Sichuan (newly recorded). **Japan:** Honshu (Aoki, 1950 [17]; Hisamatsu, 1965 [9]); Hyogo (Harusawa, 2004 [45]); Kyushu: Nagasaki (Speiser, 1928 [1]); Nara (Harusawa, 2002 [44]; Harusawa, 2004 [45]); Shikoku (Hisamatsu, 1965 [9]). **South Korea:** Gangwon-do; Gyeongsangnam-do (Young [46]).

***Grypoctonus aureus* Zhou & Li sp. nov.**

**Figure 7:**
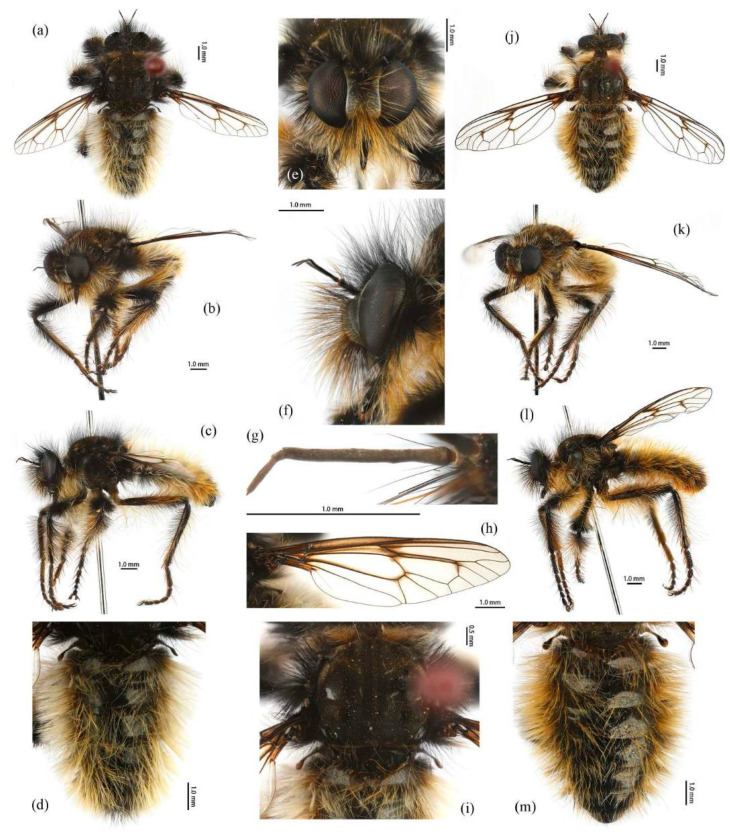
*Grypoctonus aureus* **sp. nov.**, adult. (**a**–**i**) Male; (**j**–**m**) female. (**a**) Body, dorsal; (**b**) body, profile; (c) body, lateral; (**d**) abdomen, dorsal; (**e**) head, frontal; (**f**) head, lateral; (**g**) antenna, lateral; (**h**) wing; (**i**) thorax, dorsal; (**j**) body, dorsal; (**k**) body, profile; (**l**) body, lateral; (**m**) abdomen, dorsal.

**Figure 8:**
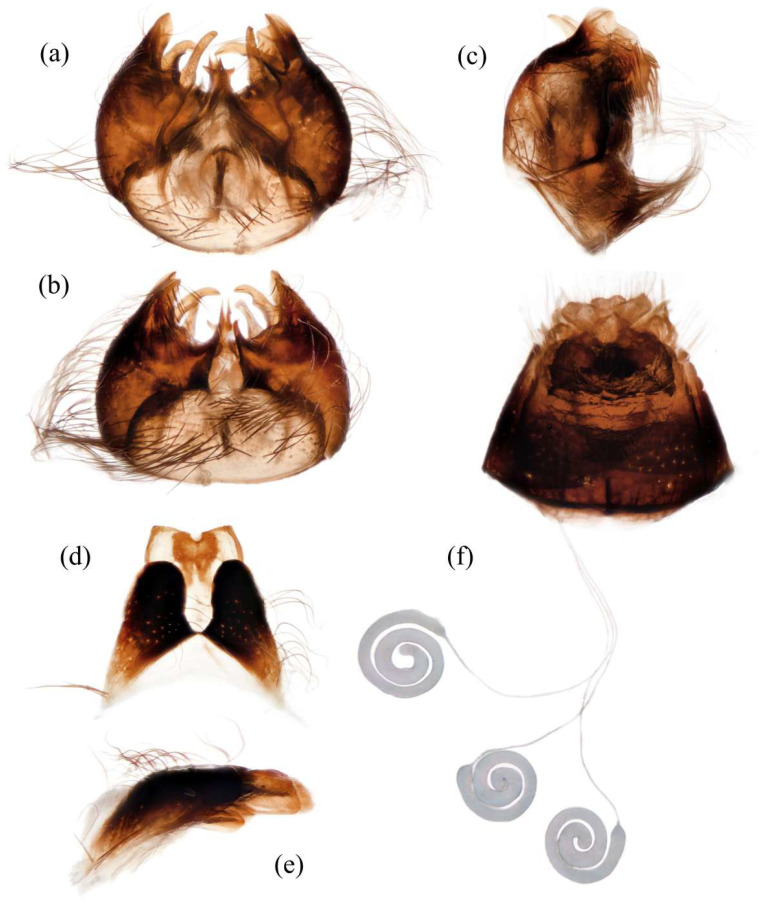
*Grypoctonus aureus* **sp. nov.**, genitalia. (**a**–**c**) Male gonocoxite and hypandrium; (**d**,**e**) cercus and epandrium. (**a**) Dorsal; (**b**) ventral; (**c**) lateral; (**d**) dorsal; (**e**) lateral; (**f**) female genitalia ventral.

Chinese common name: 错金辅脉食虫虻.

(Figure 3e–f, Figure 4e–f, Figure 7, Figure 8, and Figure 17c,d).

**Type Materials. HOLOTYPE:** ♂, **CHINA: BEIJING:** Changping [昌平], Wulisong [五里松], 40.19° N, 115.88° E, 905 m, 11.X.2024, Haoyue Zhou (CAU) (CAUDIP20243306). **PARATYPES: CHINA: BEIJING:** ♂, Yanqing [延庆], Yudushan Scenic Area [玉渡山风景区], 40.55° N, 115.88° E, 887 m, 21.IX.2023, Yuezheng Tu (CAU) (CAUDIP20231034); 4 ♂♂ 6 ♀♀, Mentougou [门头沟], Xiaolongmen National Forest Park [小龙门国家森林公园], 39.96° N, 115.43° E, 1150 m, 24.X.2023, Xuankun Li, Yuezheng Tu, Haoyue Zhou, Yan Lai (CAU) (CAUDIP20231148, CAUDIP20231165, CAUDIP20231171–CAUDIP20231178); ♂, Changping [昌平], Dade Temple [大德寺], 40.10° N, 115.54° E, 889 m, 23.IX.2024, Haoyue Zhou (CAU) (CAUDIP20243228); 4 ♂♂ 6 ♀♀, Changping [昌平], Dade Temple [大德寺], 40.10° N, 115.54° E, 889 m, 11.X.2024, Haoyue Zhou, Zhanquan Ling, Hongna Guo, Dong Guo, Tianyu Zheng (CAU) (CAUDIP20243276–CAUDIP20243285); 21 ♂♂ 27 ♀♀, Changping [昌平], Wulisong [五里松], 40.19° N, 115.88° E, 905 m, 11.X.2024, Haoyue Zhou, Zhanquan Ling, Hongna Guo, Dong Guo, Tianyu Zheng (CAU) (CAUDIP20243301, CAUDIP20243303, CAUDIP20243306–CAUDIP20243309, CAUDIP20243311–CAUDIP20243316, CAUDIP20243318, CAUDIP20243327–CAUDIP20243355, CAUDIP20243362–CAUDIP20243367). **HEBEI:** ♂, Yuxian [蔚县], Xiaowutai National Nature Reserve [小五台国家级自然保护区], 39.94° N, 114.94° E, 1123 m, 19.X.2023, Haoyang Xiong (CAU) (CAUDIP20231127). **INNER MONGOLIA:** 1 ♂ 1 ♀, Ulanqab [乌兰察布], Xinghe [兴和县], Sumu Mt. [苏木山], 40.34° N, 113.46° E, 1830 m, 4.X.2024, Zhanquan Ling (CAU) (CAUDIP20243273).

**Other Materials. CHINA: BEIJING:** ♀, Mentougou [门头沟], Huangta [黄塔], 28.IX.1984, Long Yang (NACRC, IOZ); ♂, Yanqing [延庆], Badaling [八达岭], 26.IX.1978, Yongshan Shi (NACRC, IOZ); 2 ♂♂, Yanqing [延庆], Badaling [八达岭], 5.X.1981, Zicheng Qin (NACRC, IOZ); 2 ♂♂, 2 ♀♀, Yanqing [延庆], Badaling [八达岭], 17.X.1985, Yiding Wang (NACRC, IOZ).

**Diagnosis. Male.** Medial occiput with pale yellow hairs. Pedicel with dense long black hairs and admixed with long yellow bristles. Scutum with blurry middle strip; anterolateral of transverse suture, inner-anterior of postalar callus, and the area between transverse suture and scutellum with small, bare, and shinning spots. Anterior and dorsal face of hind femora covered with yellow to pale yellow hairs and admixed with black hairs ventrally. Tergites with dense pale yellow hairs laterally. Dorsal aedeagal sheath trifurcated, branches short, with the same length. **Female.** Anepisternum densely covered with denser long yellow hairs posteriorly. Scutum with black and light yellow hairs. Anterior and dorsal face of hind femora covered with yellow hairs, ventral face of hind femora covered with black hairs on apical 1/5.

**Description. Male.** Body length 9–13 mm, wing length 16–19 mm.

**Head.** Head about 1.6× wider than high (frontal view) (Figure 7e) and 1.3× higher than long (lateral view, including face) (Figure 7f), mostly black with pale pruinescence and covered in dense long black and yellow hairs admixed with long white hairs. Frons trapezoid with light yellow pruinescence, 1.1× length of ocellar tubercle, 3.5× as wide as ocellar tubercle, with black hairs (hairs relatively short and sparse compared to hairs on other parts of the head). Ocellar tubercle slightly raised, black with sparse light yellow pruinescence and with long black hairs. Face with thick pale pruinescence and denser with long black and yellow hairs, yellow hairs longer than black hairs, without shinning strip (Figure 7e,f). Mystax not distinguishable from facial hairs. Gena narrow, with sparse pale pruinescence. Clypeus with thick light yellow pruinescence and otherwise bare. Occiput with sparse pale pruinescence and dense long black hairs, hairs denser in ventral half, hairs near the gena and the compound eyes forward bend. Antenna black with sparse light yellow pruinescence, uniform from base to apex, scape with dense long black hairs, pedicel with dense long black hairs and admixed with long yellow bristles, flagellum bare (Figure 7g). Scape 1.2× as long as wide, and 1.2× as long as pedicel; pedicel 1.1× as long as wide; flagellum 17.8× as long as wide, 2.8× as long as scape + pedicel, 5.2× as long as scape; stylus 0.3× as long as flagellum, two-segmented, with apical seta-like sensory article; apical segment 6.0× as long as basal segment. Palpus clavate, black with fine black hairs, two-segmented. Proboscis black, only slightly compressed laterally, basal 2/3 with sparse pruinescence and dense long black and light yellow hairs ventrally, apical 1/3 shinning, with short yellow hairs.

**Thorax.** Integumental color of scutum mostly black, brownish gray with dark yellow pruinescence; scutum with blurry middle strip; anterolateral of transverse suture, inner-anterior of postalar callus, and the area between transverse suture and scutellum with small, vertical, bare, and shinning spots (Figure 7i). Antepronotum covered in long yellow to white hairs; postpronotum covered in long black admixed with light yellow hairs laterally; proepimeron densely covered in long light yellow hairs. Lateral postpronotal lobe with shiny and dirty orange spot. Scutum covered with black, brownish gray pruinescence and sparse long black hairs. Scutellum black with dark brownish yellow pruinescence, covered with dense long yellow hairs. Postalar callus with long black hairs. Pleura black with thick pale pruinescence; anepisternum densely covered in long black hairs, hairs denser posteriorly, dorsally, and ventrally; katepisternum with sparse black hairs gathered on the dorsal middle area; anepimeron, katepimeron, and meron bare. Metanepisternum covered in brown and pale yellow tomentums in middle area, admixed with sparse long black hairs; metakatepisternum bare; katatergite densely covered in long black hairs dorsally and admixed with long yellow hairs; anatergite covered with dense light yellow pruinescence on dorsal margin.

**Legs.** Legs black with sparse pale pruinescence, coxae covered in dense light yellow hairs, femora mostly covered in dense long black and yellow to white hairs, tarsi with short black hairs and admixed with dark orange bristles. Anterior face of fore femora with several robust hairs on apical 1/5, posterior face of fore femora with long black hairs admixed with light yellow to white hairs, ventral face of fore femora with light yellow hairs; mid femora with black hairs admixed with light yellow hairs on anterior and ventral face on basal 3/5; anterior face of hind femora mostly covered in golden yellow hairs admixed with short black hairs, ventral face of hind femora covered with yellow hairs on basal 3/5 (Figure 4e). Fore tibiae mostly covered in dense long black hairs admixed strong long yellow bristles, ventral face of fore tibia with dense short golden yellow hairs on apical 8/9; mid tibia denser with long black hairs admixed with long dark yellow bristles; hind tibia admixed with long black and yellow hairs, posterior face on hind tibia with dense long white hairs on basal 2/5 and dense short golden yellow hairs on 3/5, admixed with long yellow hairs (Figure 3e). Ventral face of tarsi with dense short pale yellow hairs. Fore tibia 2.7× longer than fore basitarsus, mid tibia 3.3× longer than mid basitarsus, hind tibia 3.2× longer than hind basitarsus.

**Wings.** Wing membrane hyaline with infuscated wing base, cell *br*, areas around crossveins *r-m*_1_, *r-m*_2_, cell *bm,* and a half of apex of cell *d.* Haltere stem and knob brownish black (Figure 7h).

**Abdomen.** Tergites with dark blue metallic, covered with long light yellow hairs dorsally and dense pale yellow to white hairs laterally, tergites 7–8 with black hairs, tergite 7 admixed with few dark yellow hairs (Figure 7d). Sternites black with sparse pale pruinescence, covered in long black hairs admixed with pale yellow hairs; sternites 1–2 covered in pale yellow hairs admixed with black hairs. Genitalia (Figure 8a–e). Posterior margin of epandrium 1.2× longer than full length, the length between the middle point of posterior and anterior margins 0.48× longer than full length. Gonocoxite ventrally pointed on posterior margin. Basiphallus enlarged. Outer apex of gonocoxite long. Gonocoxite with large ventral extension on posterior half, trifurcated, blunt. Dorsal aedeagal sheath trifurcated, branch short, with the same length.

**Female.** Body length 11–15 mm, wing length 18–24 mm.

**Head.** Frons trapezoid with thick pale pruinescence, 3.3× as wide as ocellar tubercle. Ocellar tubercle with long light yellow hairs. Occiput with long black hairs admixed with light yellow hairs. Scape 1.6× as long as wide, and 1.3× as long as pedicel; pedicel 1.2× as long as wide; flagellum 16.4× as long as wide, 2.3× as long as scape + pedicel, 4.1× as long as scape; stylus apical segment 4.2× as long as basal segment.

**Thorax.** Scutum covered with brownish gray and dark yellow pruinescence and sparse long pale yellow hairs, admixed with short black hairs. Postalar callus with long yellow to white hairs, admixed with black hairs anteroventrally. Anepisternum densely covered in long black hairs, hairs denser dorsally and long yellow hairs posteriorly; katepisternum with sparse pale yellow hairs gathered on the dorsal middle area. Metanepisternum with several long pale yellow hairs ventrally; katatergite densely covered in long yellow hairs dorsally and admixed with black hairs anteriorly.

**Legs.** Posterior face of fore femora with yellow to white hairs, ventral face of fore femora with light yellow hairs (Figure 4f). Mid tibia denser with long black hairs admixed with long dark yellow bristles and sparse white hairs; posterior face on hind tibia with dense long pale yellow hairs (Figure 3f). Fore tibia 2.5× longer than fore basitarsus, mid tibia 4.1× longer than mid basitarsus.

**Wings.** Wing membrane hyaline with light infuscated wing base, apex of cell *br*, crossveins *r-m*_1_, *r-m*_2_, apex of cell *d* (Figure 7j).

**Abdomen.** Tergites 1–6 with black hairs antrolaterally. Sternites covered in golden yellow hairs (Figure 7m). Acanthophorite spines located on plate above cerci with 6–7 spines on each side, on plate ventrolateral of cerci with four larger spines and several smaller spines (Figure 8f).

**Remarks.** Some males have more orange hairs on tergites and legs, to the extent that they appear to belong to a different species. However, DNA barcoding confirms they are the same species, with increased orange hair being the only variation.

*G. aureus* **sp. nov.** is similar to *G. hatakeyamae*, especially the body hairs of female, but differs as follows: wing not entirely infuscated; body hairs lighter; posterior face of hind tibia with white hairs in males and pale yellow in females.

*G. aureus* **sp. nov.** is also similar to *G. lama* based on the original description. However, the type specimen of *G. lama* is lost, and its original description lacks sufficient diagnostic details. Given that *G. lama* was recorded from Qinghai, whereas *G. aureus* **sp. nov.** specimens were collected from eastern Inner Mongolia and Beijing, we propose this as a new species to accommodate the current specimens. This species was previously recorded in Beijing by Yu and Wang [42], but it was misidentified as *G. hatakeyamae*.

**Etymology.** The specific epithet “aureus” (Latin for “of gold” or “golden”) refers to the golden yellow to light yellow hairs, dark body, and metallic abdomen. The body coloration resembles the appearance of “Gold inlaying” (错金), an ancient Chinese bronze decoration technique and intangible cultural heritage. The name also alludes to the aureus, a gold coin of ancient Rome.

***Grypoctonus engeli* Hradský & Geller-Grimm, 1999**

(Figure 9).

*Grypoctonus engeli* Hradský & Geller-Grimm, 1999: 104 [3]. Type locality: India, West Bengal, Darjeeling. Holotype in ZSM, ♂.

Yatoo et al., 2024: 61 (cat.) [49].

**Figure 9:**
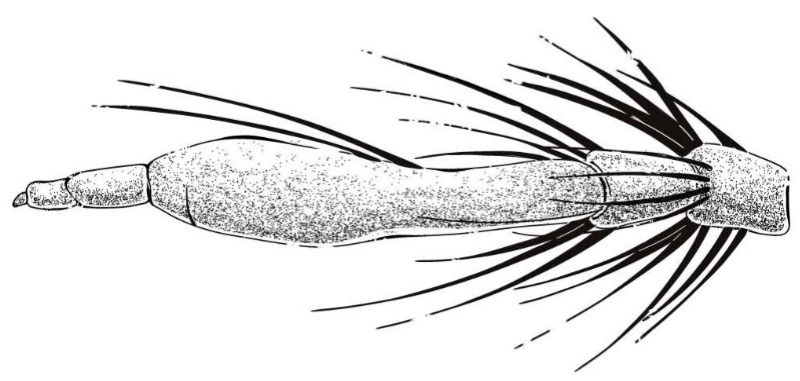
Antenna of *Grypoctonus engeli* Hradský & Geller-Grimm, 1999 (after Hradský & Geller-Grimm (1999)).

**Diagnosis.** (Modified from Hradský & Geller-Grimm, 1999 [3].) Postpedicel expanded enlarged with two black bristles (Figure 9). Scutum with light brown stripe and pale pruinescence, mesopleuron with black and white hairs. Wing membrane hyaline with infuscated anterior margin and most of crossveins. Posterior of tibia is red and anterior of tibia is black. The posterior margin of tergites 1–5 with white dense pruinescence on posterior margin and triangular in laterally. Tergite 6 with a disconnected dense pruinescence on posterior margin.

**Distribution. India:** Darjeeling (Hradský & Geller-Grimm, 1999 [3]).

***Grypoctonus hatakeyamae* (Matsumura, 1916).**

Chinese common name: 畠山辅脉食虫虻

(Figure 10).

*Grypoctonus hatakeyamae* (Matsumura, 1916): 296 (*Pycnopogon*) [2]. Type locality: Japan, Niigata [Honshu, Echigo]. Holotype ♂, paratypes 2 ♂ in EIHU.

Matsumura, 1931 (description; drawing; *Pycnopogon*) [50]; Aoki, 1950: 1600 (drawing; description; distribution; *Cyrtopogon*) [17]; Lehr, 1988: 239 (cat.; distribution) [5]; Hradský & Geller-Grimm, 1999: 105 (key; specimen information; description) [3]; Harusawa, 2002: 13 (distribution; biology) [44]; Harusawa, 2004: 67 (distribution; biology) [45]; Harusawa, 2006a: 45 (distribution; biology) [51]; Harusawa, 2006b: 51 (distribution; biology) [52]; Zhang & Yang in Yang et al., 2018: 122 (cat.) [12]; Yu & Wang, 2023: 54 (misidentification) [42].

*Grypoctonus daimyo* Speiser, 1928: 157 [1]. Type locality: Japan, Tochigi, Nikko. Syntypes in MWNH, 2 ♂ 2 ♀.

Engel, 1930: 326 (key; description) [43]; Hull, 1962: 184 (list) [7]; Lehr, 1962: 363 (photos; biology; *Cyrtopogon*) [4]; Lehr, 1964: 214 (photo; biology; *Cyrtopogon*) [19]; Lehr, 1966: 99 (distribution; biology; specimens information) [53]; Lehr, 1979: 68 (distribution; specimens information) [10]; Hradský & Geller-Grimm, 1999: 106 (as synonym of *G. hatakeyamae*; genitalia; distribution) [3]; Zhang & Yang in Yang et al., 2018: 122 (cat.; distribution) [12]; Shi, 1993: 1081 (misidentification) [11].

**Figure 10:**
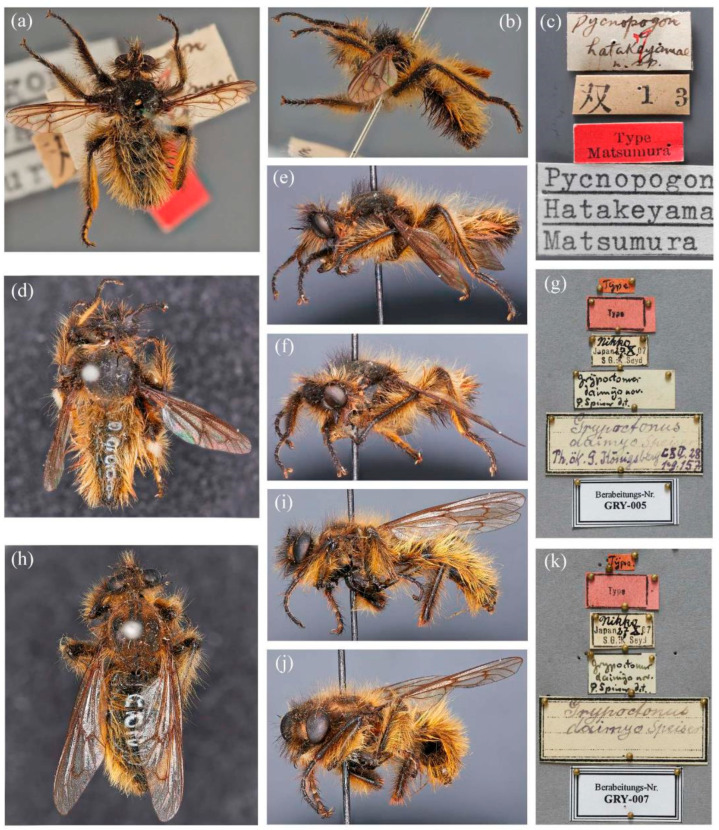
Type specimen photos of *Grypoctonus hatakeyamae* (Matsumura, 1916) (photographed by Dr. Yoko Matsumura) and *Grypoctonus daimyo* Speiser, 1928 (Photographed by Robin Kirsch). (**a**–**c**) *Grypoctonus hatakeyamae*, holotype, male; (**d**–**g**) *Grypoctonus daimyo*, syntype, male; (**h**–**k**) *Grypoctonus daimyo*, syntype, female. (**a**) Body, dorsal; (**b**) body, lateral; (**c**) label; (**d**) body, dorsal; (**e**) body, lateral; (**f**) body, profile; (**g**) label; (**h**) body, dorsal; (**i**) body, lateral; (**j**) body, profile; (**k**) label.

**Diagnosis.** Most hairs dark brown and brownish dirty yellow; wing infuscated entirely; posterior face of hind tibia covered with dark to golden yellow hairs (Figure 10).

**Distribution**. **Japan:** Gunma (Hradský & Geller-Grimm, 1999 [3]); Hyogo (Hradský & Geller-Grimm, 1999 [3]; Harusawa, 2004 [45]); Kyoto (Hradský & Geller-Grimm, 1999 [3]); Nagano (Hradský & Geller-Grimm, 1999 [3]); Niigata (Hradský & Geller-Grimm, 1999 [3]); Nikko (Engel, 1930 [43]; Hradský & Geller-Grimm, 1999 [3]); Osaka (Harusawa, 2004 [45]; Harusawa, 2006a [51]; Harusawa, 2006b [52]); **Kazakhstan:** Almaty (Lehr, 1966 [53]; Lehr, 1988 [5]); **Kyrgyzstan** (Lehr, 1966 [53]; Lehr, 1988 [5]); **Mongolia** (Lehr, 1966 [53]); **Russia**? (Lehr, 1988 [5]). **China** (questionable).

**Remarks.** Specimens of *G. hatakeyamae* were not examined in the present study; therefore, diagnosis was based on published descriptions and type photos.

There are three published records of *G. hatakeyamae* from China, but after revising these records, we consider its distribution in China to be highly questionable. Lehr (1966) [53] recorded *G. hatakeyamae* from Gansu, China, without diagnostic figures or detailed description. Considering Lehr used to treat *G. aino* as a junior synonym of *G. hatakeyamae* (Lehr, 1979) [10] and *G. aino* is known from Gansu, this record might be incorrect. Shi (1993) [11] recorded *G. hatakeyamae* from Yunnan, China. We examined the specimens and confirmed that it is an undescribed species instead of *G. daimyo* and named it *G. yongshani* **sp. nov.** (see details below). Yu & Wang [42] published a field image of *G. aureus* **sp. nov.** from Beijing but misidentified it as *G. hatakeyamae*.

***Grypoctonus lama* Speiser, 1928.**

Chinese common name: 薄辅脉食虫虻.

*Grypoctonus lama* Speiser, 1928: 156 [1]. Type locality: China, Qinghai, Qinghai Lake [Kuku-noor]. Holotype in Museum Hamburg, ♂, destroyed.

Engel, 1930: 328 (description; distribution) [43]; Hull, 1962: 184 (list) [7]; Lehr, 1988: 240 (cat.; distribution) [5]; Hradský & Geller-Grimm, 1999: 110 (key; specimen information; description) [3]; Zhang & Yang in Yang et al., 2018: 122 (cat.; distribution) [12].

**Diagnosis.** Wing hyaline, most hairs are pale, head and thorax with grayish yellow pruinescence. Most mystax yellow, only black on upper face. Abdomen entirely covered with light grayish yellow hairs, only on posterior tergite with black hairs. Hairs on the legs are lighter than *G. aino*, hind tibia with off-white hairs.

**Remarks.** The type specimen of *G. lama* is lost, leaving only the original description by Speiser [1] and a more detailed redescription by Engel [43] as references. Hradský and Geller-Grimm [3] speculated that Speiser’s specimens may have been transferred to Engel prior to the species’ description, raising doubts over whether the redescription truly represents *G. lama* or a different species. However, *G. lama* could potentially be the senior synonym of *G. aureus* **sp. nov.**, a hypothesis that could be tested if future collections from Qinghai yield specimens matching the original description.

**Distribution. China:** Qinghai (Speiser, 1928).

***Grypoctonus sagittatus* Zhou & Li sp. nov.**

**Figure 11:**
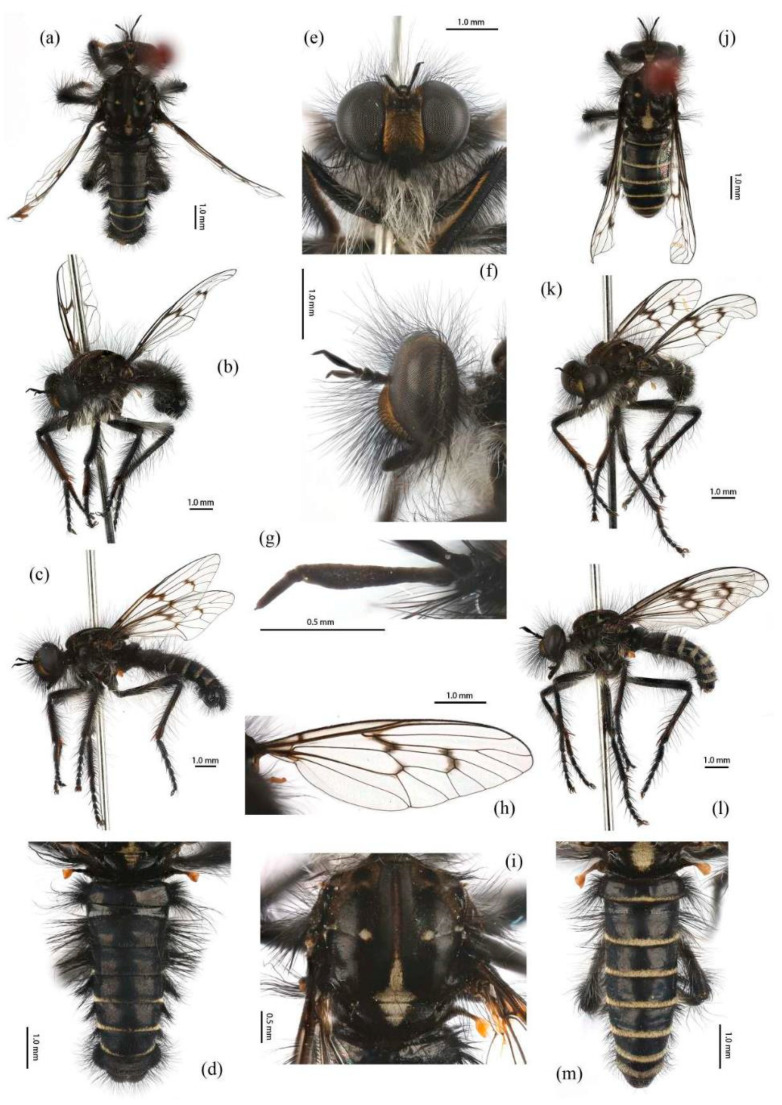
*Grypoctonus sagittatus* **sp. nov.**, adult. (**a**–**i**) Male; (**j**–**m**) female. (**a**) Body, dorsal; (**b**) body, profile; (**c**) body, lateral; (**d**) abdomen, dorsal; (**e**) head, frontal; (**f**) head, lateral; (**g**) antenna, lateral; (**h**) wing; (**i**) thorax, dorsal; (**j**) body, dorsal; (**k**) body, profile; (**l**) body, lateral; (**m**) abdomen, dorsal.

**Figure 12:**
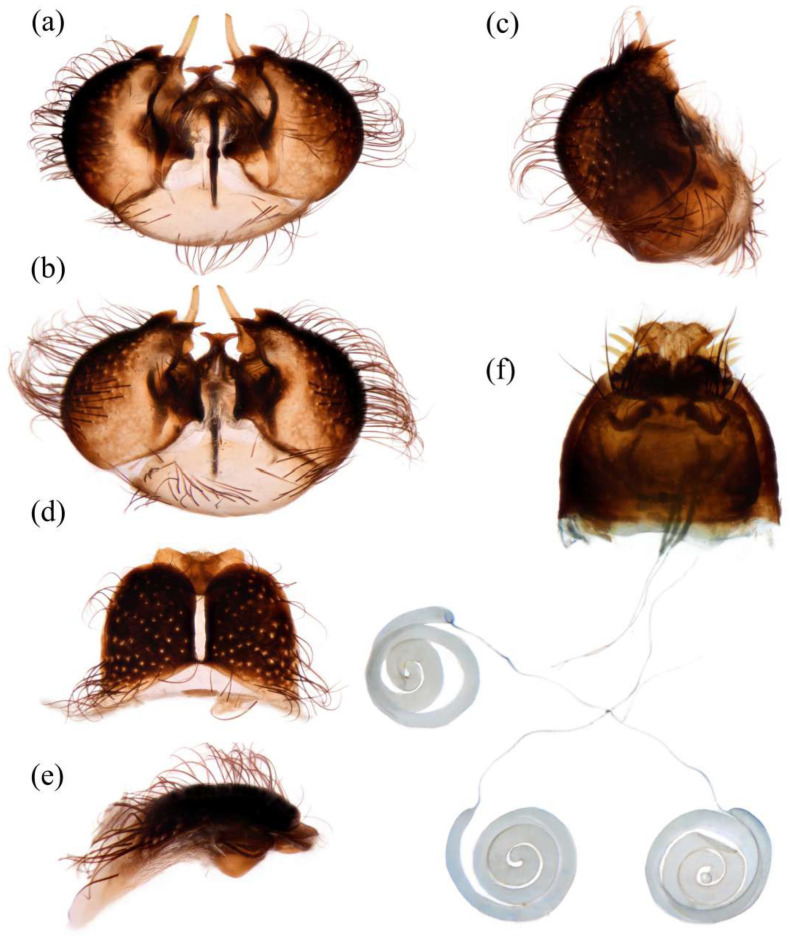
*Grypoctonus sagittatus* **sp. nov.**, genitalia. (**a**–**c**) Male gonocoxite and hypandrium; (**d**–**e**) cercus and epandrium. (**a**) Dorsal; (**b**) ventral; (**c**) lateral; (**d**) dorsal; (**e**) lateral; (**f**) female genitalia ventral.

Chinese common name: 羿箭辅脉食虫虻

(Figure 3g, Figure 4g, Figure 11, Figure 12, and Figure 17f).

**Type Materials. HOLOTYPE:** ♂, **CHINA: YUNNAN:** Baoshan [保山], Shidian [施甸], Dashuihetou Mt. [大水河头山], 24.75° N, 99.28° E, 2817 m, 14.XI.2024, Wei He (CAU) (CAUDIP20243513). **PARATYPES: CHINA: YUNNAN:** 8 ♂♂ 3 ♀♀, Baoshan [保山], Shidian [施甸], Dashuihetou Mt. [大水河头山], 24.75° N, 99.28° E, 2817 m, 14.XI.2024, Wei He (CAU) (CAUDIP20243511–CAUDIP20243512, CAUDIP20243514–CAUDIP20243517).

**Diagnosis.** Small-sized. **Male.** Apical half of postpedicel expanded. Posterior margin of scutum with inverted “T” shape pale yellow pruinescence. Scutellum with an inverted triangle pale yellow pruinescence. Hind leg with black and white hairs. Abdominal tergites 2–6 with denser white pruinescence on posterior margin. Basiphallus not enlarged. Outer apex of gonocoxite short. Dorsal aedeagal sheath trifurcated, middle branch extremely short. **Female.** Postalar callus with long black hairs and pale pruinescence. Hind femora with sparse black and pale hairs.

**Description. Male.** Body length 9–11 mm, wing length 9–15 mm.

**Head.** Head about 1.6× wider than high (frontal view) (Figure 11e) and 1.4× higher than long (lateral view, including face) (Figure 11f), mostly black with yellow and golden yellow pruinescence and covered in dense long black hairs with admixed long white hairs. Frons trapezoid with yellow pruinescence, 1.5× length of ocellar tubercle, 2.9× as wide as ocellar tubercle, with black hairs (hairs relatively short and sparse compared to hairs on other parts of the head). Ocellar tubercle slightly raised, black with sparse yellow pruinescence, with long black hairs. Face with thick golden yellow pruinescence and denser with long black hairs (Figure 11e,f). Mystax not distinguishable from facial hairs. Gena narrow, with sparse pale pruinescence. Clypeus with thick light yellow pruinescence and otherwise bare. Occiput with thick pale pruinescence and dense long black hairs, hairs denser in ventral half, hairs near the gena and the compound eyes forward bend. Antenna black with sparse light yellow pruinescence, uniform from base to apex, scape and pedicel with dense long black hairs, flagellum bare, anterior postpedicel expanded, apex of apical segment of stylus with short black hairs (Figure 11g). Scape 1.2× as long as wide, and 1.1× as long as pedicel; pedicel 1.4× as long as wide; flagellum 10.0× as long as wide, 2.3× as long as scape + pedicel, 4.5× as long as scape; stylus 0.3× as long as flagellum, two-segmented, with apical seta-like sensory article; apical segment 5.0× as long as basal segment. Palpus clavate, black with fine black hairs, two-segmented. Proboscis black, only slightly compressed laterally, basal 2/3 with sparse pruinescence and dense long black and light yellow hairs ventrally, apical 1/3 shinning, with short yellow hairs.

**Thorax.** Integumental color of scutum mostly black, brownish gray with pale yellow pruinescence; scutum with black middle strip reaching posterior 4/5 of the scutum; anterior edge of transverse suture, near the middle of scutum with round pale yellow pruinescence, surrounding it with rectangular depression; posterior margin of scutum with inverted “T” shape pale yellow pruinescence; scutellum with an inverted triangle pale yellow pruinescence (Figure 11i). Antepronotum admixed with long black and white hairs; postpronotum covered in long black admixed with white hairs laterally; proepimeron with thick pruinescence and densely covered in long white hairs. Lateral postpronotal lobe with shiny and dirty orange spot. Scutum covered with black, brownish gray with pale yellow pruinescence and sparse long black hairs. Scutellum black with sparse black and dense pale yellow pruinescence, covered with dense long black hairs. Postalar callus with long black hairs. Pleura black with thick pale pruinescence; anepisternum densely covered in long black hairs, hairs denser posteriorly and dorsally, admixed with a few white hairs; katepisternum with sparse white hairs gathered on the dorsal middle area; anepimeron, katepimeron, and meron bare. Metanepisternum densely covered in pale yellow tomentums and brownish black hairs in middle area; metakatepisternum bare; katatergite densely covered in long black hairs dorsally and long white hairs ventrally; anatergite covered with dense light yellow pruinescence on dorsal margin.

**Legs.** Legs black with sparse gray pruinescence, coxae covered in dense white hairs, femora mostly covered in dense long black and white hairs, ventral face covered in dense white hairs on basal 4/5 and black hairs on apical 1/5, apex of tibia with strong long dark orange bristles. Dorsal face of fore femora with long black hairs and mid femora with short black hairs, anterior face of fore and mid femora with several strong dark orangish to yellow bristles on middle area, all face of hind femora covered in white hairs on basal 4/5 and black hairs on apical 1/5 (Figure 3g). Fore tibiae mostly covered in dense long black hairs admixed strong long dark orange bristles, ventral face of fore tibia with dense short dark orange hairs on apical 8/9, anterior face of fore tibia with long black hairs with short dark orange bristles; mid tibia denser with long black hairs admixed with long dark orange bristles, posterior face of hind tibia with dense pale hairs on basal 2/6 to 3/6 and apical 1/6, ventral face of hind tibia with white hairs admixed with black hairs (Figure 4g). Fore tibia 2.9× longer than fore basitarsus, mid tibia 3.6× longer than mid basitarsus, hind tibia 3.1× longer than hind basitarsus.

**Wings.** Wing membrane hyaline with infuscated wing base, apex of cell *br*, posterior of cell *m*_4_, and crossveins *r-m*_1_, *r-m*_2_, and apex of cell *d*, posterior of R_4+5_. Haltere stem brownish black and knob orange (Figure 11h).

**Abdomen.** Integumental color of tergites mostly black with dark blue metallic, covered with sparse long black hairs dorsally and dense long black hairs laterally. Tergites 2–6 with white dense pruinescence on posterior margin and triangular in laterally (Figure 11d). Sternites black with sparse pale pruinescence, covered in long black hairs admixed with some white hairs; sternites 1–2 covered in white hairs admixed with black hairs. Genitalia (Figure 12a–e). Posterior margin of epandrium 1.0× longer than full length, the length between middle point of posterior and anterior margins 0.64× longer than full length. Basiphallus normal, not enlarged. Gonocoxite with large ventral extension on posterior half, bifid, blunt posteriorly, inner tapered. Outer apex of gonocoxite relatively short. Dorsal aedeagal sheath trifurcated, but the middle fork extremely short.

**Female.** Body length 8–9 mm, wing length 9–15 mm.

**Head.** Frons 1.6× length of ocellar tubercle, 2.3× as wide as ocellar tubercle. Scape 1.3× as long as pedicel; pedicel 0.7× as long as wide; flagellum 6.4× as long as wide, 2.6× as long as scape + pedicel, 4.6× as long as scape; stylus apical segment 3.0× as long as basal segment.

**Thorax.** Posterior margin of scutum with inverted “T” shape pale yellow pruinescence, extending to anterior margin of scutum and tapering gradually; proepimeron with thick white pruinescence. Postalar callus with long black hairs and with a round pale pruinescence. anepisternum densely covered in long white hairs posteriorly and admixed with long black hairs; katepisternum with thick white pruinescence dorsally.

**Legs.** Ventral face of hind tibia without white hairs. Fore tibia 2.9× longer than fore basitarsus, mid tibia 2.7× longer than mid basitarsus, hind tibia 2.9× longer than hind basitarsus.

**Wings.** Anterior and base of wing membrane more hyaline.

**Abdomen.** Tergites 1–6 with white dense pruinescence on posterior margin, with black hairs admixed with white hairs on posterolateral face (Figure 11m). Sternites 1–2 with covered in pale yellow to white hairs admixed with black hairs. Acanthophorite spines located on plate above cerci with six spines on each side, on ventrolateral plate of cerci with four larger spines and several smaller spines (Figure 12f).

**Remarks.** Tergites 2–6 of the male sometimes do not have dense white pruinescence on the posterior margin or lateral area, and such cases are usually found in the anterior and/or posterior tergites, especially tergite 2, without dense white pruinescence entirely.

*G. sagittatus* **sp. nov.** is similar to *G. engeli* but differs from it as follows: flagellum bare; dorsal face of tibia without reddish stripe.

**Etymology.** This species was named for the distinctive pattern on its scutum and scutellum, which resembles an arrow in flight accompanied by two circular markings reminiscent of celestial bodies. This imagery evokes the Chinese legend of the archer Yi, who saved the Earth by shooting down nine of ten suns. The specific epithet “sagittatus” derives from Latin “sagitta” (arrow) and the suffix “-atus” (possessing), collectively meaning “arrow-shaped”.

***Grypoctonus solarius* Zhou & Li sp. nov.**

**Figure 13:**
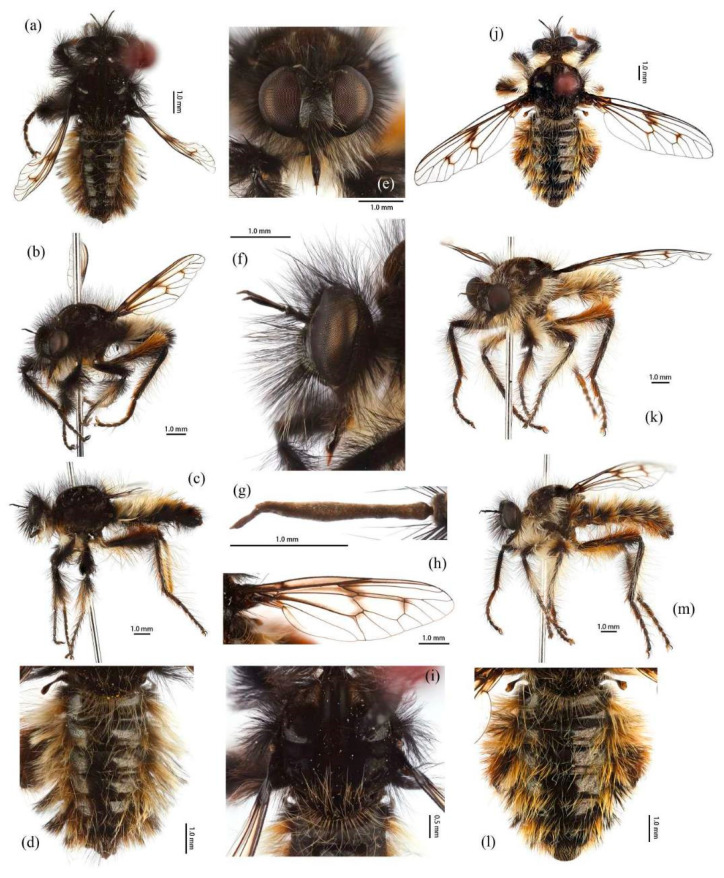
*Grypoctonus solarius* **sp. nov.**, adult. (**a**–**i**) Male; (**j**–**m**) female. (**a**) Body, dorsal; (**b**) body, profile; (**c**) body, lateral; (**d**) abdomen, dorsal; (**e**) head, frontal; (**f**) head, lateral; (**g**) antenna, lateral; (**h**) wing; (**i**) thorax, dorsal; (**j**) body, dorsal; (**k**) body, profile; (**l**) body, lateral; (**m**) abdomen, dorsal.

**Figure 14:**
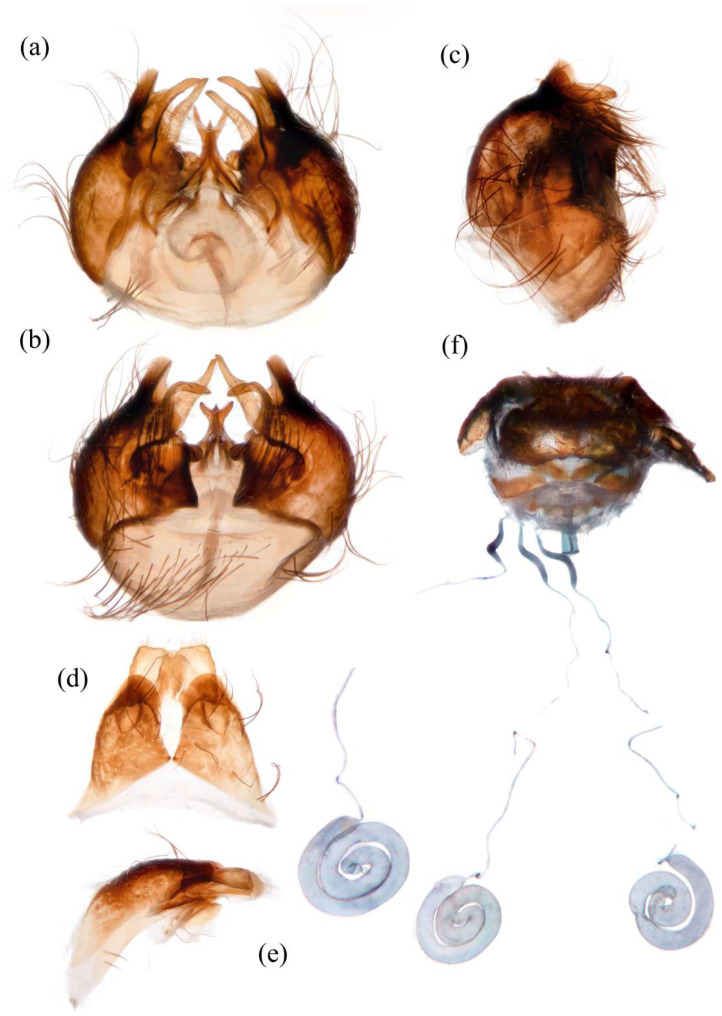
*Grypoctonus solarius* **sp. nov.**, genitalia. (**a**–**c**) Male gonocoxite and hypandrium; (**d**,**e**) cercus and epandrium. (**a**) Dorsal; (**b**) ventral; (**c**) lateral; (**d**) dorsal; (**e**) lateral; (**f**) female genitalia ventral.

Chinese common name: 秋光辅脉食虫虻

(Figure 3c,d, Figure 4c,d, Figure 13, Figure 14, and Figure 17e).

**Type Materials. HOLOTYPE:** ♂, **CHINA: YUNNAN:** Baoshan [保山], Shidian [施甸], Dashuihetou Mt. [大水河头山], 24.75° N, 99.28° E, 2817 m, 14.XI.2024, Wei He (CAU), (CAUDIP20243522). **PARATYPES:** 1 ♂ 5 ♀♀, **CHINA: YUNNAN:** Dali [大理], Jianchuan [剑川], Misha [弥沙乡], 26.27° N, 99.6° E, 3165 m, 26.X.2024, He Zhang (CAU) (CAUDIP20243471–CAUDIP20243476); 9 ♂♂ 37 ♀♀, Baoshan [保山], Shidian [施甸], Dashuihetou Mt. [大水河头山], 24.75° N, 99.28° E, 2817 m, 14.XI.2024, Wei He (CAU) (CAUDIP20243518–CAUDIP20243521, CAUDIP20243523–CAUDIP20243537).

**Other Materials. CHINA: YUNAN:** 2 ♂♂, Diqing [迪庆], Deqen [德钦], Baimang Snow Mt. [白芒雪山], 3300 m, 28.VIII.1981, Xuezhong Zhang (NACRC, IOZ).

**Diagnosis. Male.** Anterior face of hind femora covered with dark orange hairs in the top half and with black hairs in the lower half, dorsal face of hind femora with dark orange hairs, posterior face of hind tibia with dense short golden yellow hairs. Tergites with denser long black hairs ventrally. Dorsal aedeagal sheath trifurcated, middle branch short. **Female.** Anepisternum densely covered white hairs, denser posteriorly. Scutum with black and pale hairs. Anterior face of hind femora mostly covered with dark yellow hairs admixed with black hairs ventrally.

**Description. Male.** Body length 8–11 mm, wing length 13–15 mm.

**Head.** Head about 1.5× wider than high (frontal view) (Figure 13e) and 1.4× higher than long (lateral view, including face) (Figure 13f), mostly black with pale or light yellow pruinescence and covered in dense long black hairs with admixed long, white to light yellow hairs. Frons trapezoid with light yellow pruinescence, 1.3× length of ocellar tubercle, 3.2× as wide as ocellar tubercle, with black hairs (hairs relatively short and sparse compared to hairs on other parts of the head). Ocellar tubercle slightly raised, black with sparse light yellow pruinescence, with long black hairs. Face with thick pale pruinescence and admixed dense long black and a few pale hairs, except dorsolateral area with narrow, bare, and shinning strip (Figure 13e,f). Gena narrow, with sparse light yellow pruinescence. Clypeus with thick light yellow pruinescence and otherwise bare. Mystax not distinguishable from facial hairs. Occiput with thick light yellow pruinescence and dense long black hairs ventral half with admixed dense long white to light yellow hairs, light yellow hairs denser in ventral half. Antenna black with sparse light yellow pruinescence, scape, and pedicel with dense long black hairs, flagellum bare (Figure 13g). Scape 1.5× as long as wide, and 1.0× as long as pedicel; pedicel 1.4× as long as wide; flagellum 12.5× as long as wide, 2.1× as long as scape + pedicel, 4.2× as long as scape; stylus 0.2× as long as flagellum, two-segmented, with apical seta-like sensory article, apical segment 2.6× as long as basal segment. Palpus clavate, black with fine brownish to blackish hairs, two-segmented. Proboscis black, only slightly compressed laterally, basal 2/3 with sparse pruinescence and dense long pale yellow hairs ventrally, apical 1/3 shinning, with short brown to yellow hairs.

**Thorax.** Integumental color of scutum mostly brownish black with gray and light yellow pruinescence. Scutum with two brown middle strips reaching posterior 4/5 of the scutum; anterolateral of transverse suture and inner-anterior of postalar callus with small, bare, and shinning spots (Figure 13i). Antepronotum with admixed long light yellow and black hairs; postpronotum with long black hairs laterally; proepimeron densely covered in black hairs dorsally and light yellow hairs ventrally. Lateral postpronotal lobe with shiny and dirty orange spot. Scutum covered with dense brown pruinescence and sparse long black hairs. Scutellum black with sparse brown pruinescence, covered with dense long pale hairs admixed few black hairs basilally, hairs in anterior margin strongly proclinate. Postalar callus with long black hairs. Pleura black with thick brown pruinescence; anepisternum densely covered in long black hairs, hairs denser posteriorly and dorsally; katepisternum with sparse black and light yellow hairs on dorsal margin, anepimeron, katepimeron, and meron bare. Metanepisternum densely covered in light yellow tomentums and brownish black hairs in middle area, with sparse black hairs posterolaterally; metakatepisternum bare; katatergite densely covered in long black hairs; anatergite covered with dense light yellow pruinescence on dorsal margin.

**Legs.** Legs black, coxae covered in dense light yellow and white hairs, femora mostly covered in dense long black hairs, ventral face admixed with dense long light yellow hairs on basal 3/5. Dorsal face of hind femora admixed with dark orange and golden yellow hairs (Figure 4c). Fore tibiae mostly covered in dense long black hairs admixed strong long golden yellow bristles, ventral face of fore tibia with dense short golden yellow hairs on apical 4/5, posterior face of fore tibia with white hairs on apical 1/5; anterior face of mid tibia admixed with long black, dark orangish, and white hairs, dorsal face of mid tibia with half black hairs basally and half white hairs apically, admixed with long yellow hairs, posterior face on hind tibia with dense long white hairs on basal 2/5 and dense golden yellow hairs on 3/5, admixed with long yellow hairs (Figure 3f), ventral face of hind tibia with dense short golden yellow hairs. Ventral face of tarsi with dense short black and white hairs, tarsi with short black hairs admixed with dark orangish bristles and short white hairs. Fore tibia 2.7× longer than fore basitarsus, mid tibia 3.4× longer than mid basitarsus, hind tibia 3.1× longer than hind basitarsus.

**Wings.** Wing membrane hyaline with infuscated wing base, anterior margin, cell *br*, apex, and posterior of cell *bm*, areas around crossveins *r-m*_1_, *r-m*_2_, cell *r-m*, and base of cell *m*_1_. Haltere stem and knob brownish black (Figure 13h).

**Abdomen.** Integumental color of tergites mostly black with a few dark blue metallic and sparse light pruinescence. Tergite 1 with dense long pale yellow hairs, laterally admixed with more white hairs, and anterolaterally covered in dense short black hairs; tergites 2–6 with dense long pale yellow and golden yellow hairs, laterally with dense long black hairs; tergites 7–8 with long black hairs, tergite 7 admixed with few pale yellow hairs (Figure 13d). Sternites black with thick light yellow pruinescence, covered in long pale yellow hairs admixed with black hairs; sternite 1 with black hairs, admixed with pale yellow hairs, sternite 2 with black hairs, other sternites admixed with black and yellow hairs. Genitalia (Figure 14a–e). Posterior margin of epandrium 1.5× longer than full length, the length between middle point of posterior and anterior margins 0.58× longer than full length. Basiphallus enlarged. Gonocoxite with large ventral extension on posterior half, trifurcated, blunt posteriorly, inner tapered. Dorsal aedeagal sheath trifurcated, middle branch extremely short.

**Female.** Body length 9–15 mm, wing length 15–20 mm.

**Head.** Head about 1.6× wider than high (frontal view) and 1.5× higher than long (lateral view, including face). Frons 1.2× length of ocellar tubercle, 2.7× as wide as ocellar tubercle. Ocellar tubercle with pale yellow to long white hairs. Occiput with thick pale pruinescence and dense long white hairs, admixed with long black hairs, ventral half with admixed dense long black and white hairs, black hairs denser in antroventral half. Scape 1.1× as long as wide, and 0.9× as long as pedicel; pedicel 1.4× as long as wide; flagellum 10× as long as wide, 2.3× as long as scape + pedicel, 5.0× as long as scape; stylus 0.2× as long as flagellum, apical segment 1.5× as long as basal segment.

**Thorax.** Antepronotum with long pale hairs; proepimeron densely covered in white hairs. Anepisternum densely covered in long black hairs dorsally and white hairs denser posteriorly; katepisternum with sparse pale hairs on dorsal margin, katatergite densely covered in long black and light yellow to white hairs.

**Legs.** Posterior face, anterior 4/5 of ventral face of femora with light yellow to white hairs, anterior 4/5 face of mid femora with white hairs (Figure 4d). Posterior face of fore tibia with white hairs on apical 3/5 to 4/5; except ventral face of mid and hind tibia admixed with black and golden yellow hairs, other parts of mid and hind tibia mostly covered in long white hairs (Figure 3d). Fore tibia 2.8× longer than fore basitarsus, mid tibia 3.3× longer than mid basitarsus, hind tibia 3.0× longer than hind basitarsus.

**Wings.** Anterior of wing membrane hyaline, especially cell *c* (Figure 13j).

**Abdomen.** Tergites 2–7 with dense long golen yellow to pale yellow hairs, laterally with dense long black hairs; tergite 8 with pale yellow hairs. Sternite 1 with pale yellow hairs. Genitalia. Acanthophorite spines located on plate above cerci with six spines on each side, on plate ventrolateral of cerci with four larger spines and several smaller spines (Figure 14f).

**Remarks.** Apex of *R*_4+5_; some specimens slightly infuscated.

*G. solarius* **sp. nov.** is similar to *G. aino* but differs from it as follows: a dorsal face of hind femora with distinct orange hairs; male tergites with lighter hairs; and female katatergite is densely covered with long black hairs admixed with light yellow to white hairs.

**Etymology.** This species was named after its orange hairs on the anterior and dorsal face of its hind femora combined with its sun-dependent ecology. The conspicuous orange and white hairs evoke imagery of autumn landscapes bathed in golden sunlight. This fly is a solar-powered hunter, relying on daylight to pursue prey. The Latin epithet is “solarius” (meaning “solar” or “of the sun”).

***Grypoctonus yongshani* Zhou & Li sp. nov.**

**Figure 15:**
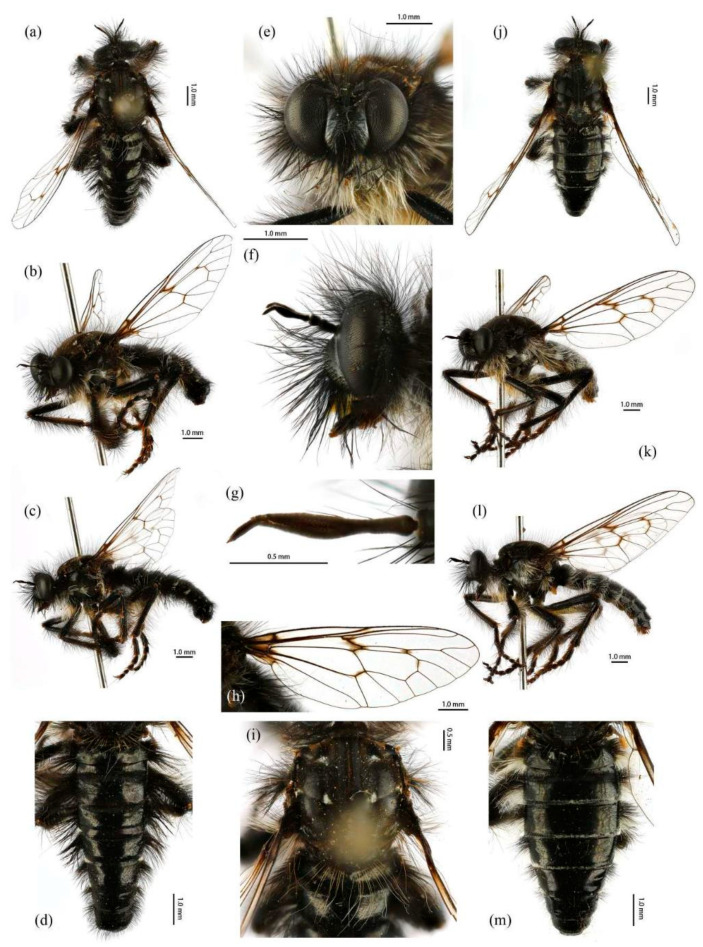
*Grypoctonus yongshani* **sp. nov.**, adult. (**a**–**i**) Male; (**j**–**m**) female. (**a**) Body, dorsal; (**b**) body, profile; (**c**) body, lateral; (**d**) abdomen, dorsal; (**e**) head, frontal; (**f**) head, lateral; (**g**) antenna, lateral; (**h**) wing; (**i**) thorax, dorsal; (**j**) body, dorsal; (**k**) body, profile; (**l**) body, lateral; (**m**) abdomen, dorsal.

**Figure 16:**
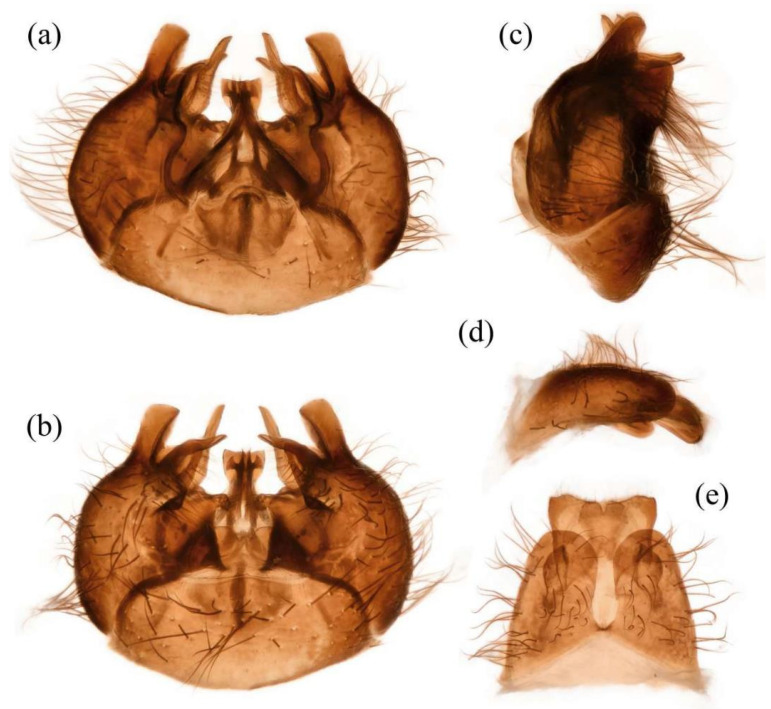
*Grypoctonus yongshani* **sp. nov.**, genitalia. (**a**–**c**) Male gonocoxite and hypandrium; (**d**,**e**) cercus and epandrium. (**a**) Dorsal; (**b**) ventral; (**c**) lateral; (**d**) dorsal; (**e**) lateral.

Chinese common name: 永善辅脉食虫虻

(Figure 3h, Figure 4h, Figure 15, and Figure 16).

**Type Materials. HOLOTYPE:** ♂, **CHINA: YUNNAN:** Diqing [迪庆], Deqen [德钦], Baimang Snow Mt. [白芒雪山], 4250 m, 31.VIII.1981, Xuezhong Zhang (NACRC, IOZ) (IOZ(E)2059369). **PARATYPES: CHINA: YUNNAN:** ♀, Diqing [迪庆], Deqen [德钦], Baimang Snow Mt. [白芒雪山], 4250 m, 31.VIII.1981, Xuezhong Zhang (NACRC, IOZ) (IOZ(E)2059370); ♀, Diqing [迪庆], Deqen [德钦], Baimang Snow Mt. [白芒雪山], 4000 m, 29.VIII.1981, Shuyong Wang (NACRC, IOZ) (IOZ(E)2059371); 2 ♀♀, Diqing [迪庆], Shangri-La [香格里拉], Xiaozhongdian [小中甸], 3800 m, 1.VIII.1984, Shuyong Wang (NACRC, IOZ) (IOZ(E)2059374–IOZ(E)2059375). **SICHUAN:** ♀, Kangding [康定], Garzê [甘孜], Gonggar Temple [贡嘎寺], 3650–4000 m, 3.IX.1982, Shuyong Wang (NACRC, IOZ) (IOZ(E)2059372); ♂, Kangding [康定], Garzê [甘孜], Gonggar Mt. [贡嘎山], 3650–4200 m, 4.Ⅸ.1982, Xuezhong Zhang (NACRC, IOZ) (IOZ(E)2059373).

**Diagnosis.** Small-sized. Apical half of postpedicel expanded, with or without two bristles. Anterior edge of transverse suture, near the middle of scutum with subtriangle pale pruinescence, surrounding it with light depression; between the scutum middle strip and lateral postpronotal lobe with subtriangle pale pruinescence. Posterior margin of scutum with short and blurred pruinescence pattern. Hind leg with black and white hairs. Dorsal aedeagal sheath blunt. Ventral aedeagal sheath four-branched, with middle two longer than later two branches. Hypandrium mammillary processes fused. **Female.** Katepisternum with thick pale pruinescence dorsally and covered in white hairs on the dorsal middle area. Notopleuron with several yellow bristles.

**Description. Male.** Body length 10–11 mm, wing length 12–15 mm.

**Head.** Head about 1.6× wider than high (frontal view) (Figure 15e) and 1.6× higher than long (lateral view, including face) (Figure 15f), mostly black with pale pruinescence and covered in dense long black hairs with admixed long white hairs. Frons trapezoid with pale pruinescence, 1.6× length of ocellar tubercle, 2.5× as wide as ocellar tubercle, with black hairs (hairs relatively short and sparse compared to hairs on other parts of the head). Ocellar tubercle slightly raised, black with sparse pale pruinescence, with long black hairs. Face with thick pale pruinescence and denser with long black hairs (Figure 15e,f). Mystax not distinguishable from facial hairs. Gena narrow, with sparse pale pruinescence. Clypeus with thick pale pruinescence and otherwise bare. Occiput with thick pale pruinescence and dense long black hairs, hairs denser in ventral half, hairs near the compound eyes forward bend. Antenna black with sparse light yellow pruinescence, uniform from base to apex, scape, and pedicel with dense long black hairs, flagellum with two hairs, anterior postpedicel expanded, apex of apical segment of stylus with short black hairs (Figure 15g). Scape 1.5× as long as wide, and 1.5× as long as pedicel; pedicel 1.0× as long as wide; flagellum 7.3× as long as wide, 2.2× as long as scape + pedicel, 3.6× as long as scape; stylus 0.2× as long as flagellum, two-segmented, with apical seta-like sensory article; apical segment 5.0× as long as basal segment. Palpus clavate, black with fine black hairs, two-segmented. Proboscis black, only slightly compressed laterally, basal 2/3 with sparse pruinescence and dense long light yellow to pale hairs ventrally, apical 1/3 shinning, with short light yellow hairs.

**Thorax.** Integumental color of scutum mostly black, brownish gray with pale yellow pruinescence; scutum with black middle strip reaching posterior 4/5 of the scutum; anterior edge of transverse suture, near the middle of scutum with subtriangle pale pruinescence, surrounding it with light depression; between the scutum middle strip and lateral postpronotal lobe with subtriangle pale pruinescence. Posterior margin of scutum with short and blurred pruinescence pattern (Figure 15i). Antepronotum with long black hairs; postpronotum covered in long black hairs laterally; proepimeron with thick pale pruinescence and densely covered in long white hairs. Lateral postpronotal lobe with shiny and dirty orange spot. Scutum covered with black, brownish gray with pale yellow pruinescence and sparse long black hairs. Scutellum black with admixed black and light yellow hairs. Postalar callus with admixed long black and light yellow hairs. Notopleuron with several yellow bristles. Pleura black with relatively thick pale pruinescence; anepisternum densely covered in long black hairs, hairs denser posteriorly and dorsally; katepisternum with thick pale pruinescence dorsally and covered with sparse white hairs gathered on the dorsal middle area, with 2–3 white hairs posteriorly and ventrally; meron with two long white hairs ventrally; anepimeron and katepimeron bare. Metanepisternum with thick pale pruinescence and densely covered in pale tomentums; metakatepisternum with thick pruinescence; katatergite densely covered with long black hairs anteriorly and posteriorly, covered in long white hairs medially; anatergite covered with dense light yellow pruinescence on dorsal margin.

**Legs.** Legs black with sparse gray pruinescence, coxae with dense pruinescence covered in dense light yellow to white hairs, femora mostly covered in dense long black and light yellow to white hairs, ventral face covered in dense light yellow to white hairs on basal 3/5 and black hairs on apical 2/5, apex of tibia with strong long orange bristles. Dorsal face of fore femora with long black hairs and mid femora with shorter black hairs, anterior face of fore and mid femora with several yellow bristles on middle area, ventral face of hind femora covered in light yellow hairs on basal 4/5 and black hairs on apical 1/5 (Figure 4h). Fore tibiae mostly covered in dense long black hairs admixed strong long dark orange bristles, ventral face of fore tibia with dense short dark orange hairs on apical 8/9, anterior face of fore tibia with long black hairs with short dark orange bristles in a row; mid tibia denser with long black hairs admixed with many long yellow bristles, posterior face of hind tibia with dense pale hairs on basal 2/6 to 3/6 and apical 1/6, other face of hind tibia with black hairs admixed with yellow bristles (Figure 3h). Fore tibia 3.7× longer than fore basitarsus, mid tibia 3.7× longer than mid basitarsus, hind tibia 3.5× longer than hind basitarsus.

**Wings.** Wing membrane hyaline with infuscated wing base, apex of cell *br*, posterior of cell *m*_4_, and crossveins *r-m*_1_, *r-m*_2_, and apex of cell *d.* Haltere stem brownish black and knob orange (Figure 15h).

**Abdomen.** Integumental color of tergites mostly black with dark blue metallic, covered with sparse long black hairs dorsally and dense long black hairs laterally. Tergites 2–6 with triangular white dense pruinescence on lateral posterior margin (Figure 15d). Sternites black with sparse pale pruinescence, covered in long black hairs admixed with some light yellow hairs; sternites 1–2 covered in black hairs. Genitalia (Figure 16a–e). Posterior margin of epandrium 1.3× longer than full length, the length between middle point of posterior and anterior margins 0.68× longer than full length. Hypandrium mammillary processes fused. Basiphallus enlarged. Lateral ejaculatory process small and fused within basiphallus. Gonocoxite with large ventral extension on posterior half, bifid, blunt posteriorly, inner slightly tapered. Outer apex of gonocoxite long. Dorsal aedeagal sheath blunt, ventral aedeagal sheath four-branched, with middle two longer than later two branches.

**Female.** Body length 10–12 mm, wing length 15–20 mm.

**Head.** Frons 2.0× length of ocellar tubercle, 2.2× as wide as ocellar tubercle. Flagellum bare. Scape 1.2× as long as wide, and 1.0× as long as pedicel; pedicel 1.2× as long as wide; flagellum 5.5× as long as wide, 4.0× as long as scape; stylus 0.6× as long as flagellum; apical segment 3.5× as long as basal segment.

**Thorax.** Posterior margin of scutum with relatively large triangular pale pruinescence. Anepisternum densely covered in long black hairs dorsally and admixed with black and white hairs posteriorly; katepisternum with thick pale pruinescence dorsally and covered in white hairs on the dorsal middle area; anepimeron, katepimeron, and meron bare. katatergite densely covered with long pale hairs, admixed with black hairs posteriorly.

**Legs.** Posterior face of fore femora with long black hairs admixed with long pale hairs. Fore tibia 2.7× longer than fore basitarsus, mid tibia 3.2× longer than mid basitarsus, hind tibia 3.2x longer than hind basitarsus.

**Wings.** Very similar to male.

**Abdomen.** Tergites 2–6 with white dense pruinescence on posterior margin and triangular in laterally (Figure 15m). Tergites 1–6 laterally with dense long black and white hairs. Sternites 1 with covered in black and pale hairs. Acanthophorite spines located on plate above cerci with six spines on each side, on plate ventrolateral of cerci with four larger spines and several smaller spines.

**Remarks.** Morphological variations were observed in several specimens, particularly in antennal vestiture and thoracic patterns. Bristles were occasionally present on flagellomeres (IOZ(E)2059371: left flagellum with one yellow bristle; IOZ(E)2059373: right flagellum with one black bristle; IOZ(E)2059375: left flagellum with two black bristles) and rarely on pedicels (IOZ(E)2059371: ventral pedicel with one yellow bristle). Additionally, thoracic patterns appeared blurred in some specimens (IOZ(E)2059371, IOZ(E)2059372, IOZ(E)2059374, IOZ(E)2059375), likely due to dust accumulation or oil contamination.

The holotype was originally identified as *Cyrtopogon daimyo* (=*Grypoctonus hatakeyamae*) by Shi [11]. Our examination revealed that this specimen does not belong to *C. daimyo* but instead represents a new species, invalidating the previous record of *C. daimyo* from Yunnan. While Shi [11] reported three specimens (1 ♂, 2 ♀♀), only two (1 ♂, 1 ♀) were found from IOZ.

*G. yongshani* **sp. nov.** is similar to *G. engli*, but the dorsal face of the tibia does not have a reddish stripe, and the scutum has special patterns.

**Etymology.** This species is named after Dr. Yongshan Shi for his important contributions to the Chinese Asilidae taxonomy.

#### 3.2.3. Key to Species of Grypoctonus

* Specimens or photographs of *G. engeli* and *G. lama* were not available for this study; therefore, their characters used were based on references only.

1. Dorsal face of tibia with reddish stripe………………………………………….***G. engeli***

– Dorsal face of tibia without reddish stripe (Figure 3)…………………………………….**2**

2. Wings hyaline, with a slight venation [according to Engel] …………………***G. lama***

– Wing subhyaline or hyaline and with infuscated wing base, vein brownish or blackish (Figure 5h, Figure 7h, Figure 10a, Figure 11h, Figure 13h, and Figure 15h)……………………………………………………**3**

3. Body hairs mostly dark brown and brownish yellow; wing infuscated entirely; posterior face of hind tibia covered with dark to golden yellow hairs (Figure 10)…………………………………………………………………………..***G. hatakeyamae***

– Body hairs mostly golden yellow or light yellow or black and white; wing hyaline with infuscated wing base; posterior face of hind tibia covered with white and yellow hairs or white and black hairs (Figure 3)…………………………….…..……**4**

4. Pedicel with yellow and black bristles; anterolateral tergites covered in dense light yellow to yellow hairs, the area between transverse suture and scutellum with a vertical, bare, and shinning spot (Figure 7c,g,i)……………………......***G. aureus* sp. nov.**

– Pedicel with only black bristles; anterolateral tergites covered in dense black hairs, the area between transverse suture and scutellum without a vertical, bare, and shinning spot (Figure 5c,i)……………………………………………..…………………**5**

5. Dorsal face of hind femora mostly covered in dark orange hairs (Figure 4c,d and Figure 13)………………………………………………………………………***G. solarius* sp. nov.**

– Dorsal face of hind femora mostly covered in black and orange or white hairs (Figure 4a,b,g,h)……………………………………………………………….…………………….**6**

6. Scutum and scutellum without pruinescence marking; posterior face of hind tibia with dense short white hairs; tergites with golden yellow hairs or black and white hairs (Figure 5i,d,m)…………………………………………….………………....***G. aino***

– Scutum or scutellum with pale pruinescence marking; posterior face of hind tibia with dense black and white hairs; tergites with only black or black and white hairs (Figure 3g,h, Figure 11i, and Figure 15i)…………………………………………………………………**7**

7. Face with thick golden yellow pruinescence; posterior margin of scutum with inverted T-shaped pale yellow pruinescence; scutellum with an inverted triangle pale yellow pruinescence (Figure 11)…………………………..…….***G. sagittatus* sp. nov.**

– Face with thick pale pruinescence; anterior edge of transverse suture, near the middle of scutum with subtriangle pale pruinescence, surrounding it with light depression; between the scutum middle strip and lateral postpronotal lobe with subtriangle pale pruinescence (Figure 13)………………………***G. yongshani* sp. nov.**

## 4. Biology

*Grypoctonus* species predominantly inhabit forests with open or semi-open canopies and vertically oriented tree trunks, an ecological preference that maximizes solar exposure during the autumn and winter months (Figure 17). Field observations reveal their strong preference for sunlit tree trunks, though individuals occasionally perch on broad leaves or exposed rocks. These flies demonstrate remarkable cold tolerance, remaining active in late autumn when most other insects are dormant and nighttime temperatures drop below freezing. They were observed to prey on hemipterans, including aphids (Aphidoidea), leafhoppers (Cicadellidae), and fleahoppers (Miridae).

## 5. Discussion

**Figure 18:**
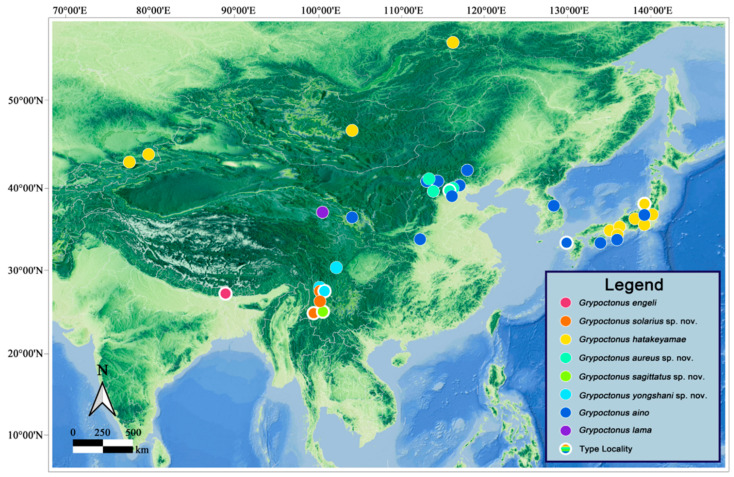
Distribution map of the genus *Grypoctonus*.

Through the comprehensive integration of current taxonomic revisions and distributional data [3,5,12,43], we recognize eight valid *Grypoctonus* species. Distribution records are present in Figure 18. This genus demonstrates remarkable biogeographic specialization, with all described species endemic to eastern Asia, adapted to Palaearctic cold temperate zones or high-elevation Oriental habitats (>2000 m). The genus exhibits unique phenological constraints, with adults’ activity strictly limited to mid-autumn through early winter. Recent collections of *G. aino* from Beijing, Hebei, Henan, Inner Mongolia, and Sichuan have bridged the previous disjunct distribution between Japanese, South Korean, and Gansu populations. Given the reduced fieldwork conducted during their active season, we suspect that substantial gaps remain in our understanding of both the distribution and species diversity of this genus.

Our study describes four new *Grypoctonus* species using integrative approaches combining morphological and molecular evidence. ABGD species delimitation analysis suggested potentially two species within *G. solarius* **sp. nov.**, with Baoshan and Dali populations forming distinct clades (Figure 1). However, comprehensive morphological comparisons of their external and genitalia characters, supported by the congruent results from ASAP, mPTP, and GMYC, confirm that these populations represent a single species. The observed genetic divergence between populations may result from insufficient taxon sampling in the present study, geographic isolation, and potential biogeographic barriers posed by the Hengduan Mountains.

## 6. Conclusions

The genus *Grypoctonus* is revised with four species newly described based on the integrated taxonomic method. Species delimitation based on COI barcodes and morphological analysis yielded congruent species hypotheses and enabled confident association of males and females for all taxa. Our results increased our knowledge of the species diversity and distribution pattern of *Grypoctonus* and substantially expanded the DNA barcodes library of this genus.

## Data Availability

The newly generated mitochondrial COI sequences are openly available on the BOLD systems under process IDs CNASI001-25 to CNASI165-25 (Supplementary File S2).

## Supplementary Materials

The following supporting information can be downloaded at https://www.mdpi.com/article/10.3390/insects16070722/s1, Supplementary File S1: Collection site information. Supplementary File S2: Information of *Grypoctonus* specimens sequenced in this study of COI sequences. Supplementary File S3: Intraspecific and interspecific genetic distance of four *Grypoctonus* species.

## References

[1] Speiser P. (1928). Kurze Kennzeichnung einer ostasiatischen Raubfliegenform. Schriften Phys.-Ökon. Ges. Königsberg.

[2] Matsumura S. (1916). Thousand Insects of Japan.

[3] Hradský M., Geller-Grimm F. (1999). Revision der Gattung Grypoctonus Speiser 1928 (Diptera: Asilidae). Mitt. Int. Entomol. Ver..

[4] Lehr P.A. (1962). Some aspects of the evolution of robber files. Tr. Inst. Zool..

[5] Lehr P.A., Soos A. (1988). Family Asilidae. Catalogue of Palaearctic Diptera.

[6] Loew H. (1847). Ueber die europaischen raubfliegen (Diptera Asilica). Linnaea Entomol..

[7] Hull F.M. (1962). Robber Flies of the World.

[8] Engel E.O. (1934). Schwedisch-chinesische wissenschaftliche Expedition nach den nordwestlichen Provinzen Chinas. II. Diptera. 3. Asilidae. Ark. Zool..

[9] Hisamatsu S. (1965). Iconographia Insectorum Japonicorum Colore Naturali Edita.

[10] Lehr P.A. (1979). Robber flies (Diptera: Asilidae) of the Amur region. Biol. Issled. Dal’n. Vost..

[11] Shi Y.S., Chen S. (1993). Diptera: Asilidae. Insects of the Hengduan Mountains Region.

[12] Zhang L.L., Yang D., Yang D. (2018). Asilidae. Species Catalogue of China.

[13] Geller-Grimm F. (2004). A world catalogue of the genera of the family Asilidae (Diptera). Stud. Dipterol..

[14] Dikow T. (2009). Phylogeny of Asilidae inferred from morphological characters of imagines (Insecta: Diptera: Brachycera: Asiloidea). Bull. Am. Mus. Nat. Hist..

[15] Cohen C.M., Noble K., Jeffrey C.T., Brewer M.S. (2021). The phylogeny of robber flies (Asilidae) inferred from ultraconserved elements. Syst. Entomol..

[16] Dikow T. (2009). A phylogenetic hypothesis for Asilidae based on a total evidence analysis of morphological and DNA sequence data (Insecta: Diptera: Brachycera: Asiloidea). Org. Divers. Evol..

[17] Aoki A., Esaki T. (1950). Family Asilidae. Iconographia Insectorum Japonicorum.

[18] Bromley S.W. (1945). The robber flies and bee-killer of China (Diptera: Asilidae). Lingnan Sci. J..

[19] Lehr P.A. (1964). On the nutrition and significance of robber flies. Tr. Inst. Zool..

[20] Loew H., Johann W.M. (1871). Beschreibungen europaischer Dipteren. Systematische Beschreibung der Bekannten Europaischen Zweiflugligen Insecten.

[21] Londt G.H., Dikow T., Ashley H. (2017). Asilidae. Manual of Afrotropical Diptera.

[22] Cumming J.M., Wood D.M., Ashley H. (2017). Adult morphology and terminology. Manual of Afrotropical Diptera.

[23] Srivathsan A., Meier R., DeSalle R. (2024). Scalable, cost-effective, and decentralized DNA barcoding with Oxford nanopore sequencing. DNA Barcoding: Methods and Protocols.

[24] Truett G.E., Heeger P., Mynatt R.L., Truett A.A., Walker J.A., Warman M.L. (2000). Preparation of PCR-quality mouse genomic DNA with hot sodium hydroxide and tris (HotSHOT). Biotechniques.

[25] Srivathsan A., Lee L., Katoh K., Hartop E., Kutty S.N., Wong J., Yeo D., Meier R. (2021). ONTbarcoder and MinION barcodes aid biodiversity discovery and identification by everyone, for everyone. BMC Biol..

[26] Puillandre N., Lambert A., Brouillet S., Achaz G.J.M.E. (2012). ABGD, Automatic Barcode Gap Discovery for primary species delimitation. Mol. Ecol..

[27] Vences M., Miralles A., Brouillet S., Ducasse J., Fedosov A., Kharchev V., Kostadinov I., Kumari S., Patmanidis S., Scherz M.D. (2021). iTaxoTools 0.1: Kickstarting a specimen-based software toolkit for taxonomists. Megataxa.

[28] Puillandre N., Brouillet S., Achaz G. (2021). ASAP: Assemble species by automatic partitioning. Mol. Ecol. Resour..

[29] Minh B.Q., Schmidt H.A., Chernomor O., Schrempf D., Woodhams M.D., Von Haeseler A., Lanfear R. (2020). IQ-TREE 2: New models and efficient methods for phylogenetic inference in the genomic era. Mol. Biol. Evol..

[30] Tavaré S. (1986). Some probabilistic and statistical problems on the analysis of DNA sequence. Lect. Math. Life Sci..

[31] Kapli P., Lutteropp S., Zhang J., Kobert K., Pavlidis P., Stamatakis A., Flouri T. (2017). Multi-rate Poisson tree processes for single-locus species delimitation under maximum likelihood and Markov chain Monte Carlo. Bioinformatics.

[32] Suchard M.A., Lemey P., Baele G., Ayres D.L., Drummond A.J., Rambaut A. (2018). Bayesian phylogenetic and phylodynamic data integration using BEAST 1.10. Virus Evol..

[33] Drummond A.J., Suchard M.A., Xie D., Rambaut A. (2012). Bayesian phylogenetics with BEAUti and the BEAST 1.7. Mol. Biol. Evol..

[34] Rambaut A., Drummond A.J., Xie D., Baele G., Suchard M.A. (2018). Posterior summarization in Bayesian phylogenetics using Tracer 1.7. Syst. Biol..

[35] Paradis E., Claude J., Strimmer K. (2004). APE: Analyses of phylogenetics and evolution in R language. Bioinformatics.

[36] Joseph V.R., Vakayil A. (2022). SPlit: An optimal method for data splitting. Technometrics.

[37] Pons J., Barraclough T.G., Gomez-Zurita J., Cardoso A., Duran D.P., Hazell S., Kamoun S., Sumlin W.D., Vogler A.P. (2006). Sequence-based species delimitation for the DNA taxonomy of undescribed insects. Syst. Biol..

[38] Tamura K., Stecher G., Kumar S. (2021). MEGA11: Molecular evolutionary genetics analysis version 11. Mol. Biol. Evol..

[39] Kimura M. (1980). A simple method for estimating evolutionary rates of base substitutions through comparative studies of nucleotide sequences. J. Mol. Evol..

[40] QGIS Development Team (2024). QGIS Geographic Information System, Version 3.40.4.

[41] Hua L.Z. (1990). Key to genera of Chinese Asilidae (II). Jiangxi Plant Prot..

[42] Yu G.Y., Wang H. (2023). Asilidae. The Beijing Forest Insect Atlas—Diptera.

[43] Engel E.O., Lindner E. (1930). Asilidae. Die Fliegen der Paläarktischen Region.

[44] Harusawa K. (2002). Observations of the oviposition and hatching of *Grypoctonus aino* Speiser, 1928 and *G.hatakeyamae* (Matsumura, 1916) (Diptera:Asilidae). Hana Abu.

[45] Harusawa K. (2004). Records of prey of the genus *Grypoctonus* (Diptera: Asilidae), and notes on Bibionidae (Diptera) as their prey. Hana Abu.

[46] Young C.L. (2025). Robber flies of South Korea—I. South Korean species of the Subfamily Stenopogoninae Hull (Diptera, Asilidae). Stud. Dipterol..

[47] Tagawa Y. (2020). Asilidae in Japan. http://www3.kcn.ne.jp/~tgw/web_page/Stenopogoninae/Grypoctonus%20aino-e.htm.

[48] Bock K., Mengual X. (2023). New records of Japanese robber flies (Diptera: Asilidae) deposited at the ZFMK collections. Bonn Zool. Bull..

[49] Yatoo S.F., Wachkoo A.A., Pillai K.G., Cohen C.M. (2024). Catalog of the Robber Flies of India (Diptera: Asilidae). Zootaxa.

[50] Matsumura S. (1931). 6000 Illustrated Insects of The Empire of Japan.

[51] Harusawa K. (2006). Manual for the study robber files (Diptera: Asilidae). Hana Abu.

[52] Harusawa K. (2006). An observation of the courtship behavior of *Grypoctonus hatakeyamae* (Matsumura) (Diptera:Asilidae). Hana Abu.

[53] Lehr P.A. (1966). Biology and taxonomy of robber flies (Diptera, Asilidae) of the genera *Cyrtopogon* Loew and *Grypoctonus* Speiser of Kazakhstan and of Middle Asia. Biol. Geogr..

